# TRIM33 Is a Co-Regulator of Estrogen Receptor Alpha

**DOI:** 10.3390/cancers16050845

**Published:** 2024-02-20

**Authors:** Bianca A. Romo, Barbara Karakyriakou, Lauren Cressey, Brooke L. Brauer, Huijuan Yang, Alexa Warren, Anneka L. Johnson, Arminja N. Kettenbach, Todd W. Miller

**Affiliations:** 1 Department of Molecular and Systems Biology, Geisel School of Medicine at Dartmouth, Lebanon, NH 03755, USA; 2 Department of Biochemistry and Cell Biology, Geisel School of Medicine at Dartmouth, Lebanon, NH 03755, USA; 3 Department of Pharmacology and Toxicology, Medical College of Wisconsin, Milwaukee, WI 53226, USA; 4 Department of Pathology, Medical College of Wisconsin, Milwaukee, WI 53226, USA

**Keywords:** ER+ breast cancer, estradiol, hormone signaling, transcription cofactor, protein stability

## Abstract

**Simple Summary:**

Dysregulation of estrogen receptor alpha (ER) activity has been implicated in the development of resistance to current treatment strategies. The purpose of this study was to profile novel coregulatory proteins to identify potential therapeutic targets. We identified TRIM33 as a novel interactor and regulator of ER activity and protein stability. These results provide rationale for further investigation of the role of TRIM33 in cancers exhibiting increased ER protein levels.

**Abstract:**

Estrogen receptor alpha (ER)-positive breast cancer is responsible for over 60% of breast cancer cases in the U.S. Among patients diagnosed with early-stage ER+ disease, 1/3 will experience recurrence despite treatment with adjuvant endocrine therapy. ER is a nuclear hormone receptor responsible for estrogen-driven tumor growth. ER transcriptional activity is modulated by interactions with coregulators. Dysregulation of the levels of these coregulators is involved in the development of endocrine resistance. To identify ER interactors that modulate transcriptional activity in breast cancer, we utilized biotin ligase proximity profiling of ER interactomes. Mass spectrometry analysis revealed tripartite motif containing 33 (TRIM33) as an estrogen-dependent interactor of ER. shRNA knockdown showed that TRIM33 promoted ER transcriptional activity and estrogen-induced cell growth. Despite its known role as an E3 ubiquitin ligase, TRIM33 increased the stability of endogenous ER in breast cancer cells. TRIM33 offers a novel target for inhibiting estrogen-induced cancer cell growth, particularly in cases of endocrine resistance driven by ER (*ESR1*) gene amplification or overexpression.

## 1. Introduction

Breast cancer is one of the leading diagnosed cancers in women in the United States, affecting approximately 30% of all female cancer patients [1]. Breast cancer is subtyped based on molecular profiling for the expression of hormone receptors [progesterone receptor (PR), estrogen receptor alpha (ER)] and human epidermal growth factor receptor 2 (HER2). Among diagnosed patients, 2/3 of breast cancer cases are ER-positive (ER+) [2]. Patients afflicted with ER+ breast cancer typically receive endocrine therapies that target the estrogen/ER signaling axis to mitigate its growth-promoting effects.

ER+ breast tumor growth is induced by estrogen steroid hormones, including 17-beta-estradiol (E2). ER is a transcription factor encoded by the gene *ESR1* that, upon binding of estrogen ligand, will homodimerize and be shuttled into the nucleus. ER will then bind to estrogen response elements (EREs) within DNA to modulate gene expression [3]. As an added layer of gene regulation, liganded ER will undergo conformational changes exposing helix 12, allowing for binding of coregulatory proteins that modulate ER transcriptional activity [4,5,6,7,8]. Coregulators involved in ER transcriptional activation include p300, SRC-3, BRG-1, CARM1, and TET2 [9,10,11,12,13,14,15]. In a paradoxical manner, E3 ubiquitin ligases, most commonly known for their role in protein degradation, have coactivator abilities in the context of ER signaling: ubiquitin–protein ligase E3A (UBE3A, E6AP) interacts with ER in a ligand-dependent manner and localizes to EREs in DNA alongside ER [16,17]; mouse double minute 2 homolog (MDM2) promotes the expression of the ER-inducible gene *TFF1* by co-binding with the ER in the *TFF1* promoter [17].

Transcriptional dysregulation can lead to the development of resistance to endocrine therapies. One-third of patients with ER+ breast cancer being treated with adjuvant endocrine therapies experience recurrence within 15 years of initial diagnosis [18]. One such mechanism of resistance is alteration of ER coregulator levels. Homeobox B7 (HOXB7), a mediator of DNA damage checkpoint 1 (MDC1), and Octamer-binding transcription factor 4 (OCT-4) are co-regulators shown to modulate ER function and contribute to the development of endocrine resistance [19,20,21]. Endocrine-resistant ER+ breast cancer cells showed increased expression of HOXB7 that, when in complex with ER, drives the expression of ER target genes and HER2 (another driver of endocrine resistance) [19]. In the presence of the selective ER modulator tamoxifen, OCT-4 directed ER binding to genomic loci that led to a gene expression profile associated with cell proliferation [21].

We aimed to enhance the understanding of potential contributors to the development of therapeutic resistance in ER+ breast cancer. To identify novel interactors that modulate ER activity, we utilized the technique of proximity-dependent biotin labeling with TurboID [22]. This system uses a mutant biotin ligase from *E. coli*, biotin ligase (BirA), that has undergone yeast display-directed evolution, resulting in the 35-kD biotin ligase TurboID [23]. The TurboID method avoids confounding toxicity issues encountered with other methods for proximity-dependent labeling that rely on enzymes like horseradish peroxidase (HRP) or engineered ascorbate peroxidase (APEX) [23,24]. Additionally, HRP is limited in its ability to carry out its labeling function depending upon cellular location, limiting its use for intracellular environments [25]. The benefits of using this updated BioID system allows for labeling of transient and weak interactions in minutes rather than hours without affecting cell viability, providing a record of interactions as the ligase moves through a cell [26,27,28]. Using the proximity labeling capabilities of TurboID, a TurboID-ER fusion construct was generated to profile ER interactomes. Profiling identified tripartite motif containing 33 (TRIM33, TIF1γ) as an interactor with ER. TRIM33 promoted ER transcriptional activity and stabilized the ER protein, supporting an oncogenic role for TRIM33 in ER+ disease.

## 2. Results

### 2.1. Proximity Labeling of ER Identifies TRIM33 as an Interactor

**Figure 1:**
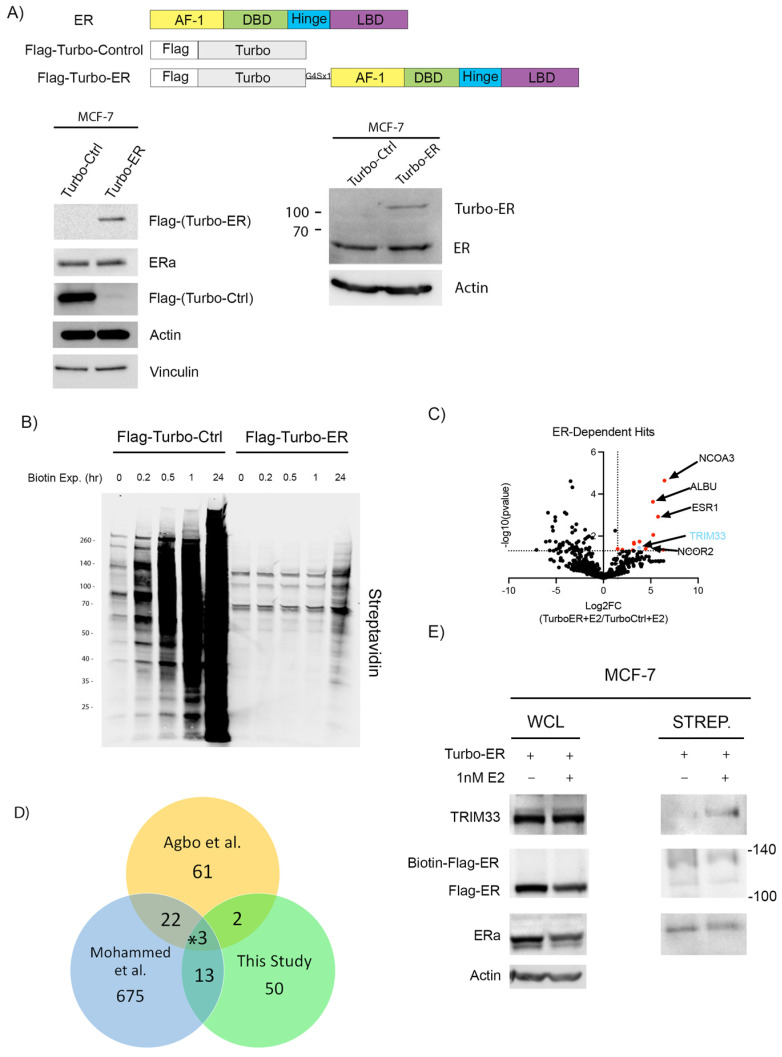
Estrogen-induced proximity labeling of ER interactors. (**A**) Construct maps of wild-type ER, Flag-TurboID-Control (Turbo-Ctrl), and Flag-TurboID-ER (Turbo-ER). Lysates from MCF-7 cells stably transduced with Turbo-Ctrl or Turbo-ER were analyzed by immunoblot. (**B**) Cell lines from (**A**) were exposed to 100 μM of biotin for 0–24 h. Lysates were analyzed by immunoblot that was probed with streptavidin-conjugated dye. (**C**) Volcano plot of results from LC-MS/MS analysis of streptactin pulldowns from cell lines from (**A**) treated with 1 nM of E2 for 1 h. Interactors significantly (*p* ≤ 0.05) enriched in Turbo-ER samples compared to Turbo-Ctrl are highlighted as colored dots. Blue dot indicates TRIM33. Log2FC: log_2_(fold-change). (**D**) Venn diagram of numbers of ER interactors identified in three studies ([29,30] and this study). * TRIM33. (**E**) Lysates from MCF-7/Turbo-ER cells treated with +/− E2 for 24 h were used for streptactin pulldown followed by immunoblot. WCL: whole cell lysate. File S1: Original western blots.

To begin profiling the interactome of ER, we generated an N-terminally TurboID-labeled ER construct (Turbo-ER) and an unconjugated TurboID control (Turbo-Ctrl). These constructs were used to generate stable cell lines using MCF-7 human ER+ breast cancer cells (Figure 1A). The use of TurboID-labeled proteins can influence the function of endogenous proteins if the fusion construct is expressed at a significantly higher level. However, transduction with Turbo-ER did not substantially alter the endogenous levels of ER (Figure 1A).

Validation was performed to determine the biotinylation capabilities of both constructs over a period of 24 h. The control construct showed promiscuous biotinylation ability with saturation occurring as early as 10 min after biotin treatment. Turbo-ER did not reach such levels of saturation even after 24 h (Figure 1B). To ascertain the early interactors associated with ER upon E2 supplementation, E2 and biotin were administered for 1 h and protein was subsequently harvested. These studies revealed 68 proteins that were significantly (*p* ≤ 0.05) enriched in E2-treated MCF-7/Turbo-ER cells compared to the MCF-7/Turbo control (Figure 1C and Table 1). Among the statistically significant ER interactor hits identified herein, molecular functions revealed by gene ontology analysis include nuclear steroid receptor activity, chromatin binding, and transcription coregulator activity (Figure S1), all of which pertain to canonical ER signaling. Comparing our findings of an ER interactome with those previously reported in ER+ breast cancer cells [29,30], only three ER interactors were common to all studies (NCOA3, NCOR2, and TRIM33; Figure 1D). While the effects of NCOA3 (AIB1, SRC3) and NCOR2 (SMRT) on ER are well known [31,32], the effects of TRIM33 on ER have not yet been reported. In conjunction with TRIM33 being identified by all three interactomes, further support for investigating the role for TRIM33 in ER+ breast cancer comes from findings in prostate cancer cells where TRIM33 modulates the activity of another nuclear hormone receptor, the androgen receptor [33]. In validation experiments, proximity labeling was performed with MCF-7/Turbo-ER cells treated with +/− E2 for 24 h. Biotinylation of TRIM33 was appreciably higher in E2-treated cells (Figure 1E), suggesting an estrogen-induced interaction of Turbo-ER with TRIM33.

### 2.2. TRIM33 Possesses ER Regulatory Capabilities

**Figure 2:**
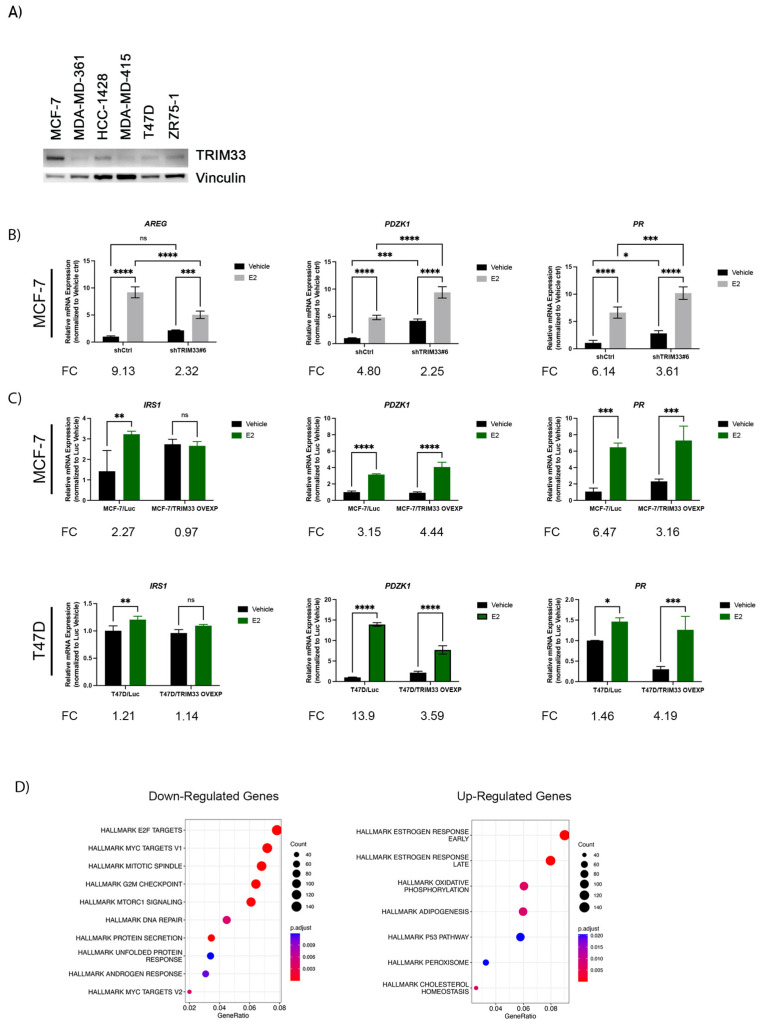
TRIM33 Regulates ER Signaling. (**A**) Immunoblot of lysates from ER+ breast cancer cell lines. (**B**) RT-qPCR of ER target genes in MCF-7/shCtrl and MCF-7/shTRIM33#6 cells treated with +/− 1 nM E2 for 24 h. E2-induced fold change (FC) relative to vehicle treatment is shown below each chart. (**C**) RT-qPCR of ER target genes in MCF-7 and T47D cells overexpressing luciferase (Luc) control or TRIM33 treated with +/− 1 nM E2 for 24 h. Data in (**B**,**C**) are shown as mean of triplicates + SD. * *p* ≤ 0.05, ** *p* ≤ 0.01, *** *p* ≤ 0.001, **** *p* ≤ 0.0001 by Bonferroni multiple comparison-adjusted post-hoc test. ns: not significant. (**D**) RNA-seq was performed using MCF-7/shCtrl and MCF-7/shTRIM33#6 cells treated with 1 nM E2 for 24 h. Sets of genes downregulated or upregulated by TRIM33 knockdown were analyzed for enrichment of Hallmark pathways. Number of genes in a pathway is reflected by circle size. Proportion of genes in a pathway is indicated along x-axis. Adjusted *p*-value of enrichment for a pathway is indicated by color. File S1: Original western blots.

Since the interaction of TRIM33 with ER is enhanced by E2 (Figure 1E), we tested TRIM33’s effects on ER function in an estrogen-replete setting. A comparison of ER+ breast cancer cell lines showed that TRIM33 levels were highest in MCF-7 (Figure 2A); thus, MCF-7 cells were used to generate two stable shRNA knockdown polyclonal models (#5 and #6, Figure S2). Conversely, T47D cells had relatively low TRIM33 expression (Figure 2A), prompting their use as a model for overexpression of exogenous TRIM33 (Figure S3). While E2 increased the expression of canonical ER-inducible transcripts in MCF-7/shCtrl cells, TRIM33 knockdown blunted these effects (Figure 2B). These results indicate that TRIM33 plays a role in the regulation of E2-induced ER signaling. However, TRIM33 overexpression did not uniformly promote ER activity, exhibiting variable effects across canonical E2/ER-inducible genes and cell lines (Figure 2C). To avoid biased results due to gene selection, we performed transcriptome-wide RNA-seq analysis of MCF-7/shCtrl and MCF-7/shTRIM33 knockdown cells treated with +/− E2 for 24 h (Figure S4). Hierarchical clustering of the 10% most differentially expressed genes showed that replicate samples clustered together based on (i) E2 treatment and (ii) the shRNA construct. Hallmark pathway analysis of RNA-seq profiles showed that the set of genes downregulated by shTRIM33 during E2 treatment was enriched for pathways related to DNA repair, the G2M checkpoint, and E2F Targets (Figure 2D), pathways in line with known effects of TRIM33 [33,34,35,36,37]. The set of genes upregulated by shTRIM33 during E2 treatment was enriched for estrogen response pathways (Figure 2D); these observations parallel the RT-qPCR results showing elevated levels of select ER-inducible transcripts (*PDZK1*, *PR*) with TRIM33 knockdown upon supplementation with E2 (Figure 2B). These results suggest a role for TRIM33 in the control of E2/ER-driven signaling.

**Figure 3:**
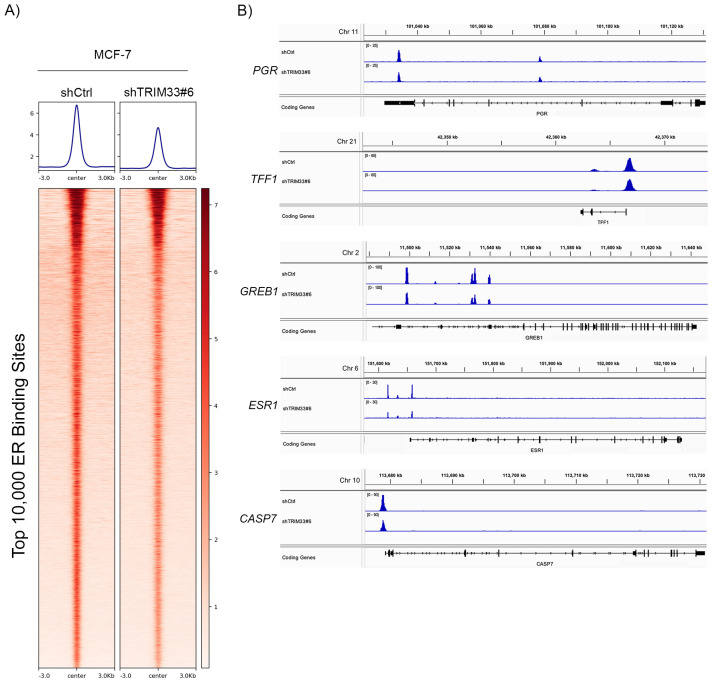
TRIM33 Alters ER Binding to Chromatin. (**A**) MCF-7/shCtrl and MCF-7/shTRIM33#6 cells were treated with +/− 1 nM E2 for 24 h and then analyzed by ChIP-seq for ER. Heatmaps show the top 10,000 ER binding sites as measured by peak intensity +/− 3 kb of peak center. (**B**) ChIP-seq profiles within *PGR*, *TFF1*, *GREB1*, *ESR1*, and *CASP7* genomic regions bound by ER. Chromosome coordinates are based on hg38 annotation.

We obtained additional direct evidence of TRIM33’s effects on ER transcriptional activity by genome-wide profiling of ER binding sites (“cistromes”) using chromatin immunoprecipitation with DNA sequencing (ChIP-seq). TRIM33 knockdown generally decreased E2-induced ER binding to chromatin in MCF-7 cells (Figure 3A). Examination of canonical ER binding sites within the ER-regulated genes *TFF1*, *GREB1*, *PGR*, *CASP7,* and *ESR1* (encodes ER) revealed decreased ER binding upon TRIM33 knockdown (Figure 3B). These results suggest a role for TRIM33 in directing ER binding to chromatin.

### 2.3. TRIM33 Stabilizes ER Protein Levels

**Figure 4:**
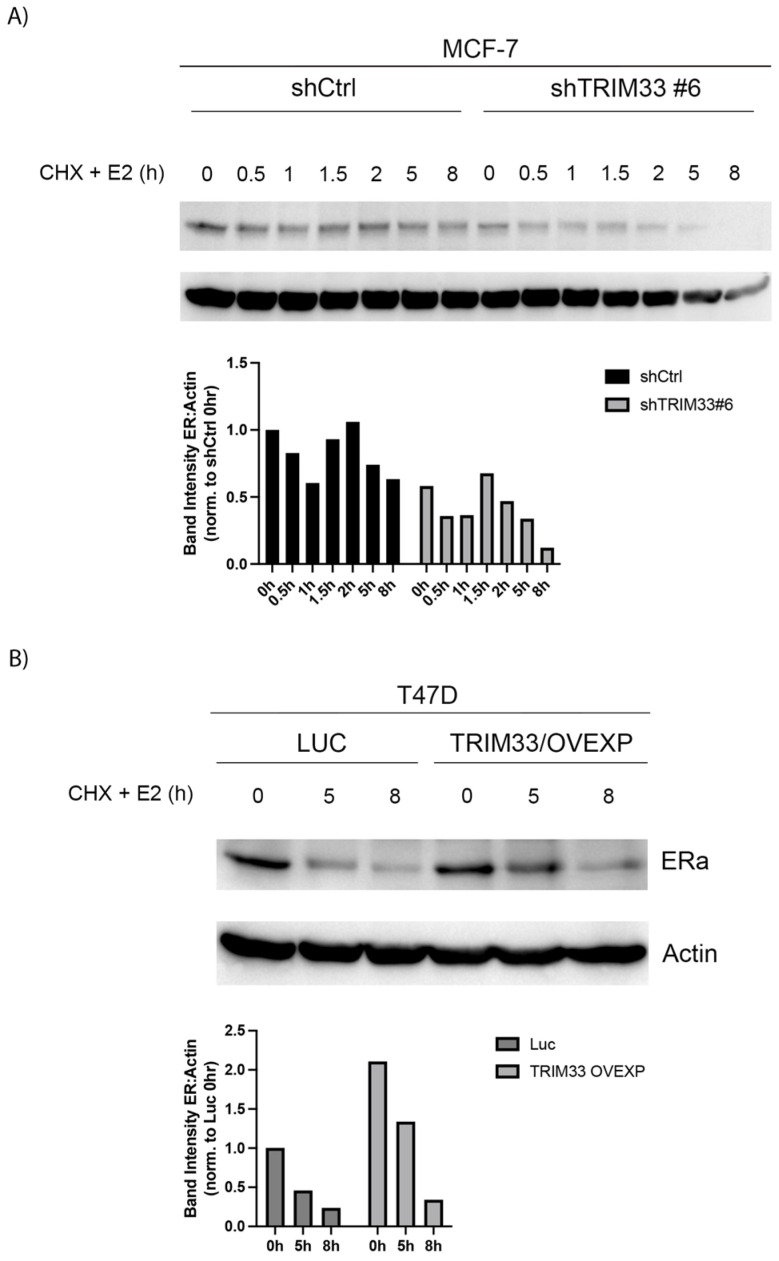
TRIM33 Stabilizes ER Levels. (**A**) MCF-7/shCtrl and MCF-7/shTRIM33#6 cells were hormone deprived for 4 d and then treated with 1 nM E2 and 100 μM cycloheximide for 0–8 h. Lysates were analyzed by immunoblot. (**B**) T47D/Luc and T47D/TRIM33 OVEXP cells were treated and analyzed as in (**A**). File S1: Original western blots.

Since transcription factor levels can affect apparent transcriptional output, we tested the effects of TRIM33 on ER levels. TRIM33 knockdown decreased ER protein levels in hormone-depleted conditions (Figure 4A). Estrogens induce ER degradation via the proteasome [38]. In cycloheximide pulse-chase assays, TRIM33 knockdown exacerbated E2-induced ER turnover. Conversely, TRIM33 overexpression prevented E2-induced ER turnover in T47D cells (Figure 4B). These data collectively indicate that TRIM33 stabilizes ER.

### 2.4. TRIM33 Regulates E2-Driven ER+ Breast Cancer Cell Growth

**Figure 5:**
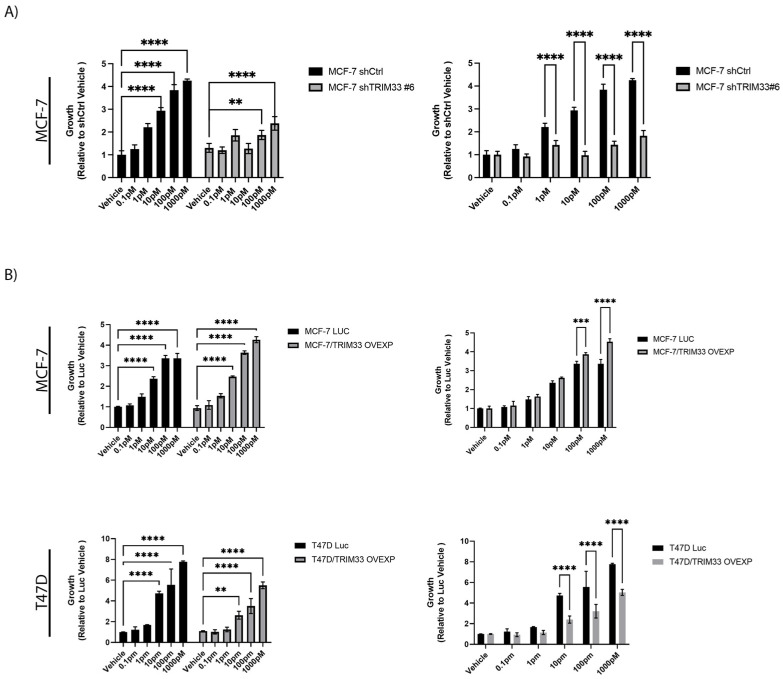
TRIM33 Modulates E2-Driven Cell Growth. (**A**) MCF-7/shCtrl and MCF-7/shTRIM33#6 cells were hormone deprived for 4 d and then treated with 0–1 nM of E2 for 7 d before analysis. (**B**) MCF-7 and T47D cells overexpressing luciferase control (Luc) or TRIM33 were treated and analyzed as in (**A**). Data shown are mean of triplicates + SD. ** *p* ≤ 0.01, *** *p* ≤ 0.001, **** *p* ≤ 0.0001 by Bonferroni multiple comparison-adjusted post-hoc test. Left column depicts growth relative to vehicle-treated shCtrl (**A**) or Luc (**B**) cells. Right column depicts growth relative to each vehicle-treated control.

TRIM33 has proposed roles as an oncogene or tumor suppressor in breast cancer [39,40]. We found that ER+ breast cancer cells with TRIM33 knockdown remained growth induced by E2 stimulation (Figure 5A and Figure S5). However, in examining the fold change in growth induced by E2, we observed a significant desensitization to E2 upon TRIM33 knockdown. This desensitization parallels RNA-seq results showing TRIM33 knockdown-induced reduction in genes involved in the G2/M cell cycle and E2F pathways despite E2 stimulation (Figure 2D). However, TRIM33 overexpression showed cell line-dependent effects on growth (Figure 5B). MCF-7 cells overexpressing TRIM33 remained sensitive to E2 stimulating effects only at higher doses. Conversely, T47D/TRIM33 OVEXP cells were growth stimulated by E2 but overall showed slower growth compared to the T47D/Luc control. These results indicate that TRIM33 promotes the growth of MCF-7 cells in an E2-dependent manner. Among the cell lines tested, MCF-7 cells possessed the highest steady-state levels of TRIM33 expression, while T47D exhibited relatively low levels of expression (Figure 2A). It should be considered that the cell line-dependent responses to TRIM33 overexpression might lie in different tolerances to TRIM33 levels.

## 3. Discussion

ER+ breast cancer is typically driven by the mitogenic effects of estrogens. To identify co-regulators that can modulate ER transcriptional activation, we used the biotin ligase TurboID to profile an estrogen-induced ER interactome in breast cancer cells. We identified TRIM33 as an E2-inducible interactor of ER that modulates ER stability and transcriptional activity. Comparing our ER-interacting hits with those of two prior reports [29,30], TRIM33 was detected in all three interactomes. Although each of these three studies identified numerous ER interactors, limited overlap was observed between interactomes (Figure 1D). Such differences may be explained by differing techniques (biotin labeling vs. rapid immunoprecipitation mass spectrometry of the endogenous protein [RIME] that captures proteins in complex with chromatin), duration of E2 signal induction, and biological model systems. Additionally, the two prior studies profiled the ER interactome under steady-state conditions, while experiments performed in this study were conducted under hormone-deprived conditions and subsequent stimulation with E2 to capture early protein interactions. The prior TurboID-based study [30] allowed for prolonged biotin labeling, which could have contributed to the differences in the proteins identified.

TRIM33 (TIF1γ) is a member of the transcriptional intermediary 1 (TIF1) family of proteins involved in chromatin binding. Across several cancer types, TRIM33 has been shown to have varied roles as a tumor suppressor or oncogene [34,41,42,43,44], suggesting a context-dependent function of TRIM33. TRIM33 has been implicated in the progression and prognosis of liver, pancreatic, lung, and prostate cancers acting in a context-dependent manner as an oncogene or tumor suppressor [33,44,45,46]. A meta-analysis across several ER+ breast cancer datasets using the KM plotter [47] showed no association of tumor *TRIM33* mRNA levels with patient survival. In breast cancer, conflicting results confound the classification of TRIM33 as an oncogene or tumor suppressor [39,40,48]. One report cites TRIM33 levels as being decreased in breast tumors relative to normal breast tissue. TRIM33-mediated destabilization of the SMAD3/SMAD4 complex was shown to inhibit TGF-β-driven metastasis [40]. Another report associated tumor TRIM33 overexpression with a poor prognosis in relation to the TGF-β pathway [39]. These results highlight the context- and expression-dependent nature of the TRIM33 function.

In preclinical models of ER+ breast cancer, we found that TRIM33 plays a role(s) in the regulation of E2-driven signaling and growth, but the effects varied between cell lines and levels of response. At the genomic level, TRIM33 knockdown in MCF-7 cells generally decreased ER binding to canonical genomic loci (Figure 3), suggesting that TRIM33 may be required for ER activity. At the protein level, TRIM33 stabilized ER in MCF-7 and T47D cells (Figure 4). E2 induces ER activation, ubiquitination, and proteasomal degradation, providing a negative feedback circuit to modulate global ER activity. In addition, the ER function is partially regulated by protein turnover, and proteasomal inhibition negatively impacts ER transcriptional activation [8]. Thus, if TRIM33 inhibits ER degradation, ER transcriptional activation may be compromised. At the mRNA level, TRIM33 knockdown increased basal and E2-induced ER activity at canonical ER-inducible genes and increased enrichment for transcriptional profiles reflecting an estrogen response (Figure 2B,D). TRIM33 overexpression did not increase E2-induced signaling (Figure 2), suggesting that endogenous TRIM33 levels may saturate signaling. At the cellular level, TRIM33 knockdown desensitized MCF-7 cells to E2 (Figure 5), but TRIM33 overexpression did not elicit a growth advantage. T47D cells were growth inhibited by TRIM33 overexpression, which may be due to T47D cells having relatively low basal levels of TRIM33 that are optimal for their growth. As an ER-interacting protein, the gene-specific role(s) of TRIM33 requires further in-depth analysis to determine the seemingly disparate effects on ER signaling and the E2 growth response.

A limitation of this study is that we did not visualize the direct interaction of ER with TRIM33 (i.e., by co-immunoprecipitation), possibly due to a transient nature of this interaction. While we did not pinpoint the molecular mechanism by which TRIM33 modulates ER activity, we postulate that this occurs via altered protein degradation or transcriptional regulation. TRIM33 is canonically known as an E3 ubiquitin ligase, and such ligases typically promote proteasomal degradation of target proteins. However, our data suggest that TRIM33 stabilizes ER protein levels (Figure 4). TRIM33 was recently reported to have stabilizing effects on androgen receptors in prostate cancer cells by preventing Skp-2 mediated proteasomal degradation [33]. The androgen receptor and ER are similar nuclear hormone receptors driving endocrine-related cancers, raising the possibility that TRIM33 has similar stabilizing effects on both targets.

Extending our findings to potential explanations for mechanisms of endocrine resistance, it has been observed that elevated levels of ER can promote resistance to estrogen deprivation and even promote therapeutic sensitivity to estrogen treatment [49,50]. For patients with ER+ breast cancer exhibiting high levels of TRIM33 and ER, TRIM33 inhibition may be considered a therapeutic opportunity to downregulate ER and prevent estrogen-independent tumor growth, as is observed upon resistance to aromatase inhibitor therapy. Using long-term estrogen deprived (LTED) MCF-7 cells with acquired resistance to hormone deprivation as a model for resistance to aromatase inhibitors, we observed relatively higher TRIM33 expression compared to MCF-7 controls. TRIM33 knockdown suppressed the hormone-independent growth of MCF-7/LTED cells, supporting TRIM33’s use as a potential therapeutic target. E3 ligase-targeting drugs are under development [51,52,53]. Current methodologies for E3 targeting aim to inhibit enzymatic activity, disrupt substrate binding, and modulate protein expression [54]. Further investigation of the dynamics of the interaction between TRIM33 and ER are necessary to rationally develop targeted therapies. Due to the apparent context-dependent nature of TRIM33 and its widespread expression across tissues, TRIM33-directed therapy may require targeting of cancer cells to avoid toxicity to normal tissues.

## 4. Materials and Methods

### 4.1. Cell Culture

MCF-7 and T47D cell lines were obtained from the American Type Culture Collection (ATCC) (Manassas, VA, USA). LentiX cells were obtained from Clontech. Cells were cultured in Dulbecco’s Modified Eagle’s Medium (DMEM; Corning) with 10% fetal bovine serum (FBS; R&D Systems, Minneapolis, MN, USA). For experiments conducted in hormone-deprived conditions, cells were cultured for the specified time in phenol red-free DMEM (Corning) supplemented with 10% dextran-coated charcoal-treated FBS (DCC-FBS; R&D Systems) and 2 mM of GlutaMAX (ThermoFisher Scientific, Waltham, MA, USA).

### 4.2. Plasmids and Cloning

Plasmid pHAGE-ESR1 was a gift from Gordon Mills and Kenneth Scott (Addgene plasmid # 116737, Watertown, MA, USA) and was used for cloning of wild-type *ESR1*. pLenti PGK V5-LUC Puro (w543-1) was a gift from Eric Campeau and Paul Kaufman (Addgene plasmid # 19360) and underwent restriction digestion with SalI and XbaI to remove luciferase and was used as a backbone for plasmid generation below. Primers used for plasmid construction are listed in Table S2. Plasmid pCMV10-3XFLAG-TurboID [55] was used to obtain the 3XFLAG–TurboID sequence. To generate plasmid Flag-Turbo-Control: 3XFLAG-TurboID was cloned into digested (as above) pLenti PGK V5 (w543-1) plasmid backbone using Hifi DNA assembly Master Mix (New England Biolabs, Ipswich, MA, USA). To generate plasmid Flag-Turbo-ER: the *ESR1* sequence was cloned from pHAGE-ESR1 plasmid for fusion with 3XFLAG-TurboID (Flag-Turbo-ER) using Hifi DNA assembly Master Mix and inserted into pLenti PGK V5 (w543-1). ER was labeled at the N-Terminus to avoid affecting the C-terminal ligand binding domain of ER.

*TRIM33* cDNA was cloned from plasmid HA-TRIM33_pInducer20 (synthesized by Genescript) into pLenti PGK V5 (as above). Lentiviral vectors encoded constitutively active shRNA targeting TRIM33 (sh#5 catalog # V2LHS-134255, and sh#6 catalog # V2LHS-134259) from Dharmacon or non-targeting shRNA control (catalog # SHC002) from Sigma-Aldrich.

Lentivirus was produced using Lentix cells with transfer plasmids described above (Flag-TurboID-ER, shRNA ctrl, shTRIM33#6, shTRIM33#5), pMD2.G (#12259), and psPAX2 (#12260). pMD2.G and psPAX plasmids were gifts from Didier Trono and obtained from Addgene. Transduced cells were selected with puromycin (1 μg/mL) for 7 d.

### 4.3. Immunoblotting

Chemicals were purchased from Sigma unless noted. Cells were rinsed twice with PBS, then lysed using RIPA buffer (20 mM Tris pH 7.4, 150 mM NaCl, 1% NP-40, 10% glycerol, 1 mM EDTA, 1 mM EGTA, 5 mM NaPPi, 50 mM NaF, 10 mM Na β-glycerophosphate) plus fresh HALT protease inhibitor cocktail (Pierce, Waltham, MA, USA) and 1 mM Na_3_VO_4_ (New England Biolabs). Lysates were sonicated at 30% power for 10 s, then centrifuged at 17,000× *g* for 10 min at 4 °C. Protein content was quantified from lysate supernatant by bicinchoninic acid (BCA) assay (Pierce), and lysates were diluted to equivalent protein concentrations across samples. Protein extracts were reduced with 1.25% β-mercaptoethanol and denatured with 4X NuPAGE buffer (ThermoFisher Scientific) and heating to 95 °C for 1 min. Proteins were separated using SDS-PAGE and transferred to nitrocellulose membranes. Membranes were probed with antibodies targeting ER (1:1000, Santa Cruz #SC-8002, Santa Cruz, CA, USA), TRIM33 (1:500, Cell Signaling Technology #90051, Danvers, MA, USA), β-actin (1:5000, Cell Signaling Technology #3700), vinculin (1:5000, Cell Signaling Technology #13901), FLAG (1:1000, Millipore Sigma #F3165, Burlington, MA, USA), and IRDye 800CW Streptavidin (1:3000, LI-COR Biosciences #926-32230, Lincoln, NE, USA). Signal was detected via horseradish peroxidase-conjugated secondary antibodies (GE Healthcare, Chicago, IL, USA) and developed with ECL substrates (Pierce). An 800CW-tagged antibody was visualized using the LI-COR odyssey imaging system.

### 4.4. Cycloheximide Assay

Cells were hormone deprived for 3 d and reseeded. On Day 4, cells were treated with 100 μM of cycloheximide and 1 nM of E2 over an 8 h time course. At specified time points, lysates were harvested for immunoblot.

### 4.5. RT-qPCR

Cells were hormone deprived for 3 d and reseeded. On Day 4, cells were treated with +/− 1 nM E2 for 24 h. RNA was harvested using RNeasy Universal Plus Mini Kit (Qiagen, Germantown, MD, USA). RNA was reverse transcribed with iScript cDNA Synthesis kit (Bio-Rad, Hercules, CA, USA). Real-time qPCR was performed with iQ SYBR Green SuperMix (Bio-Rad) with a Bio-Rad CFX96 thermocycler. Data were analyzed by ΔΔCT method. Primer sequences are listed in Table S1.

### 4.6. Growth Assay

Cells were hormone deprived for 3 d and reseeded. On Day 4, cells were treated with 0–1 nM of E2. On Day 11, cells were fixed with 10% trichloroacetic acid for 30 min at 4 °C. Wells were washed with water and allowed to dry. Wells were stained with 0.4% sulforhodamine B (SRB) for 10 min. Wells were rinsed with 1% acetic acid and allowed to dry. SRB was solubilized with 10 mM of Tris (pH 7.5), and signal was measured at an absorbance of 490 nm via microplate reader.

### 4.7. Biotinylated Protein Pulldown

For mass spectrometry assays, cells were hormone deprived for 8 d and treated for 1 h with 100 μM biotin +/− 1 nM E2. For immunoblot assays, cells were hormone deprived for 4 d and treated with 100 μM biotin +/− 1 nM E2 × 24 h. Cells were rinsed with PBS and lysed with base lysis buffer (50 mM Tris pH 7.5, 500 mM NaCl, 0.5% Triton X-100, 5 mM β-glycerophosphate, 2 mM NaF, 2 mM molybdate) with 1:500 Protease Inhibitor Cocktail (Research Products International, Mt Prospect, IL, USA). Lysates were centrifuged at 17,000× *g* for 10 min at 4 °C. Protein concentrations were determined by BCA assay (Pierce), and concentrations were equalized across samples. Strep-Tactin Sepharose 50% suspension beads (IBA Lifesciences, Goettingen Germany) were used for biotinylated protein pulldown. Lysate was incubated with beads for 3 h at 4 °C. Beads were washed and protein was eluted in 100 μL 2% SDS, 50 mM Tris pH 8.0, and 5 mM biotin at 85 °C for 15 min. Eluted protein was analyzed by LC-MS/MS (described below) or immunoblot.

### 4.8. Label-Free LC-MS/MS Analysis

Proximity-labeled samples were analyzed on a Q-Exactive Plus quadrupole Orbitrap mass spectrometer (ThermoScientific) equipped with an Easy-nLC 1000 (ThermoScientific) and nanospray source (ThermoScientific). Peptides were resuspended in 5% methanol/1% formic acid and loaded onto a trap column (1 cm length, 100 μm inner diameter, ReproSil, C18 AQ 5 μm 120 Å pore (Dr. Maisch, Ammerbuch, Germany)) vented to waste via a micro-TEE and eluted across a fritless analytical resolving column (35 cm length, 100 μm inner diameter, ReproSil, C18 AQ 3 μm 120 Å pore) pulled in-house (Sutter P-2000, Sutter Instruments, San Francisco, CA, USA) with a 45 min gradient of 5–30% LC-MS buffer B (LC-MS buffer A: 0.0625% formic acid, 3% ACN; LC-MS buffer B: 0.0625% formic acid, 95% ACN).

The Q-Exactive Plus was set to perform an Orbitrap MS1 scan (R = 70K; AGC target = 1 × 10^6^) from 350 to 1500 *m*/*z*, followed by HCD MS2 spectra on the 10 most abundant precursor ions detected by Orbitrap scanning (R = 17.5K; AGC target = 1 × 10^6^; max ion time = 50 ms) before repeating the cycle. Precursor ions were isolated for HCD by quadrupole isolation at width = 1 *m*/*z* and HCD fragmentation at 26 normalized collision energy (NCE). Charge state 2, 3, and 4 ions were selected for MS2. Precursor ions were added to a dynamic exclusion list +/− 20 ppm for 15 s. Raw data were searched using COMET (release version 2014.01) in high-resolution mode [56] against a target–decoy (reversed) [57] version of the human proteome sequence database (UniProt; downloaded February 2020; 40,704 entries of forward and reverse protein sequences) with a precursor mass tolerance of +/− 1 Da and a fragment ion mass tolerance of 0.02 Da, and requiring fully tryptic peptides (K, R; not preceding P) with up to 3 miscleavages. Static modifications included carbamidomethylcysteine and variable modifications included oxidized methionine. Searches were filtered using orthogonal measures including mass measurement accuracy (+/− 3 ppm), Xcorr for charges from +2 to +4, and dCn targeting < 1% FDR at the peptide level. Quantification of LC-MS/MS spectra was performed using MassChroQ [58] and the iBAQ method [59]. Missing values were imputed from a normal distribution in Perseus to enable statistical analysis [60].

### 4.9. RNA-Seq

Cells were hormone deprived for 4 d and then treated with +/− 1 nM of E2 for 24 h. RNA was harvested from biological triplicates using RNeasy Universal Plus Mini Kit (Qiagen). RNA was quantified by Qubit, and integrity was measured on a fragment analyzer (Agilent, Santa Clara, CA, USA). RNA (200 ng) was hybridized to FastSelect probes (Qiagen) for ribo-depletion, followed by library preparation using RNA HyperPrep kit (Roche, Basel, Switzerland). Libraries were pooled for sequencing on a NextSeq2000 (Illumina, San Diego, CA, USA) to obtain 30M paired-end 50 bp reads/sample. Sequencing reads were trimmed for low-quality reads and adapter contamination with Cutadapt [61]. Trimmed reads were aligned using STAR aligner (v2.7.1a) and indexed with SAMtools index [62,63]. Raw gene read counts were generated using featureCounts (v2.0.5) [64]. Differential expression analysis was performed using DESeq2 (V1.41.0) [65].

### 4.10. ChIP-Seq

Fifteen million cells per sample were hormone deprived for 4 d and treated with 1 nM of E2 for 1 h. Biological duplicates were fixed by adding 10% of the culture volume of 11% formaldehyde into media for 10 min at room temperature, and the reaction was quenched with 2.5 M of glycine at 5% of the culture volume. Cells were collected by scraping, centrifuged at 4 °C at 1350× *g* for 5 min, washed twice with cold PBS, flash frozen with liquid nitrogen, and stored at −80 °C overnight. Cells were then thawed and resuspended in 30 mL of cold lysis buffer 1 (50 mM HEPES-KOH pH 7.5, 140 mM NaCl, 1 mM EDTA, 10% glycerol, 0.5% NP-40, 0.25% Triton X-100), incubated at 4 °C for 10 min, pelleted, then lysed again in 30 mL cold lysis buffer 2 (10 mM Tris-HCl pH 8, 200 mM NaCl, 1 mM EDTA pH 8, 0.5 mM EGTA) at 4 °C for 10 min. After pelleting, cells were resuspended in sonication buffer (50 mM HEPES-KOH pH 7.5, 140 mM NaCl, 1 mM EDTA, 1 mM EGTA, 1% Triton X-100, 0.1% Na-deoxycholate, 1% SDS) and sonicated with a Covaris E220 to shear chromatin to a 500 bp average length. Ten percent of the sonicated volume was set aside as “input sample”. The remaining sheared chromatin was diluted in sonication buffer without SDS to yield a final SDS concentration ≤ 0.1%.

Bead–antibody complexes were prepared by mixing ChIP-Grade Protein G Magnetic Beads (Cell Signaling Technology) with ER antibody (Cell Signaling Technology #8644) at 0.079 mg/mL concentration and rotating at 4 °C overnight. Bead–antibody complexes were washed and resuspended with cold blocking buffer (0.5% BSA in PBS), and then bead suspension was added to sonicated chromatin and IP was performed by rotation at 4 °C overnight. Bead–antibody–chromatin mix was washed 3 times with blocking buffer. Immunoprecipitates were tagmented using the ChIPmentation procedure [66]. DNA was eluted and crosslinks (including those with input samples) were reversed at 65 °C overnight. Eluted DNA was treated with RNase A and Proteinase K and then purified using Quick-DNA Microprep Kit (Zymo Research, Irvine, CA, USA).

Eluted immunoprecipitated DNA and input DNA were amplified with 11 PCR cycles using custom barcoded primers (Nextera, Juno Beach, FL, USA), quality control checked on a Fragment Analyzer (Agilent, Santa Clara, CA, USA), quantified by Qubit, and pooled for sequencing on a NextSeq2000 (Illumina). Libraries were sequenced using 50 bp paired-end reads to obtain 50 M reads per sample. Sequencing reads were processed with the ENCODE Histone ChIP-seq pipeline (v2.2.1) (github.com/ENCODE-DCC, Cambridge, MA, USA) and aligned to the human genome (hg38) using Bowtie2 (v2.5.1) with minimum read length set at 2 [67]. Unmapped/low-quality reads were filtered using SAMtools (v1.16.1), and duplicated reads were removed using Picard (v2.27.5).

### 4.11. ChIP-Seq Quantification and Statistical Analysis

ChIP-seq fragment lengths were estimated with cross-correlation analysis; enriched regions were identified, and signal tracks with ChIP fold-enrichment over-input scores were generated using MACS2 (v2.2.7.1) with *p*-value threshold set to 0.01 [68]. The deepTools (v3.5.2) computeMatrix tool was used to create a matrix of the top 10,000 peak signals and plotted using deepTools plotHeatmap [69]. Peaks were visualized by plotting signal over tracks on IGV (v2.13.1).

### 4.12. Statistical Analysis

For proximity labeling experiments, statistical analysis was carried out in Perseus by two-tailed Student’s *t*-test. Growth assays and RT-qPCR results were analyzed by ANOVA with Bonferroni-adjusted post-hoc testing between groups. Growth assays, RT-qPCR, and immunoblots experiments were repeated on a minimum of two occasions.

## 5. Conclusions

Through proximity-dependent labeling techniques, we identified the E3 ubiquitin ligase TRIM33 as an estrogen-driven interactor of ER. Our results indicate that TRIM33 plays a role in regulation of estrogen-dependent ER function via modulation of transcriptional activity and protein stability. Our work highlights the rationale for further investigation of the role of TRIM33 in the progression of cancers characterized by increased levels of ER expression. 

## Figures and Tables

**Table 1:** Significant E2-Induced Interactors of ER. Proteins are identified by gene symbol and grouped based on identification in one or more interactome studies noted in Figure 1D.

Identified in all 3 studies:							
TRIM33	NCOA3	NCOR2					
Identified in 2 studies:							
KMT2D	ESR1	RAI1	RXRB	FUBP3	U2SURP	G3BP1	MCCC2
IDH1	KDM1A	SNRPD3	CCT7	AHSA1	SMARCD2	PCNP	
Identified only in this study:							
PC	CD9	ALB	TFAP2A	ETFB	TLE4	CBX3	SNRPB
SNRPN	SNRPD2	SYNCRIP	SERBP1	CCT4	TJP1	EEF1D	PIP4K2C
NIBAN2	CSRP1	UBAP2L	SP100	HMGB1P1	SAFB	GSPT2	ILF3
KIF5C	ERP44	NDUFA10	KIF5A	NUP153	KIF5B	PGD	PRCC
RAB27A	CRIP2	SELENBP1	CAPZA2	FHL1	RXRG	CAPZA1	L1RE1
ATP5MF	MYL12A	CALU	SEPTIN2	GRB2	PAK3	RAB27B	MYL12B
TAGLN3	HNRNPAB						

## Data Availability

RNA-seq data and ChIP-seq data are available at NCBI SRA under accession # PRJNA1027811.

## Supplementary Materials

The following supporting information can be downloaded at https://www.mdpi.com/article/10.3390/cancers16050845/s1, Figure S1—Enriched Molecular Functions of ER Interactome Proteins; Figure S2—Generation of TRIM33 shRNA Knockdown Cell Lines; Figure S3—Generation of TRIM33-overexpressing Cell Lines; Figure S4—Transcriptomic Changes induced by TRIM33 Knockdown; Figure S5—TRIM33 Knockdown with Separate Construct (#5) Suppresses E2-Stimulated Growth; Table S1: Primers for RT-qPCR; Table S2: Primers for Generation of Flag-Turbo-ESR1 and TRIM33/OVEXP Constructs. Original western blots are presented in File S1.
