# Supplementary material for: TRIM33 Is a Co-Regulator of Estrogen Receptor Alpha
Source: Cancers (Basel). 2024 Feb 20;16(5):845. doi: 10.3390/cancers16050845 (PMC10930732; doi:10.3390/cancers16050845)
Supplement: Supplementary file 1 [file cancers-16-00845-s001.zip › cancers-2855756-supplementary.pdf]

cancers

Supplemental Data for Romo et al.

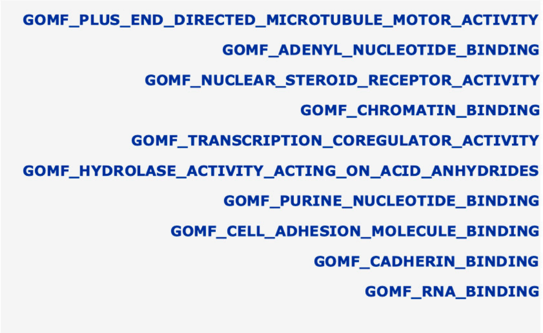

Figure S1. Enriched Molecular Functions of ER Interactome Proteins. Proteins identified in the ER interactomes of the current study were used for Molecular Signatures Database (MSigDb) analysis to identify enriched molecular functions using default parameters.

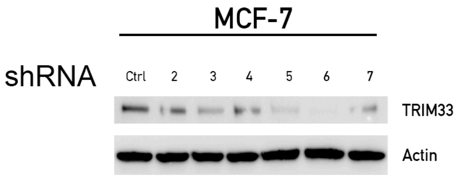

Figure S2. Generation of TRIM33 shRNA Knockdown Cell Lines. MCF-7 cells were stably transduced with shRNA targeting TRIM33 (6 different constructs) or non-silencing control. Lysates were analyzed by immunoblot.

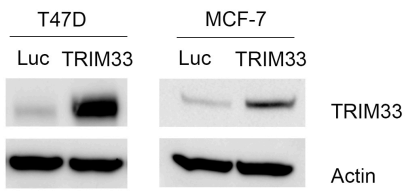

Figure S3. Generation of TRIM33-overexpressing Cell Lines. MCF-7 and T47D cell lines were stably transduced with vectors encoding Luc or TRIM33. Expression was confirmed by immunoblot.

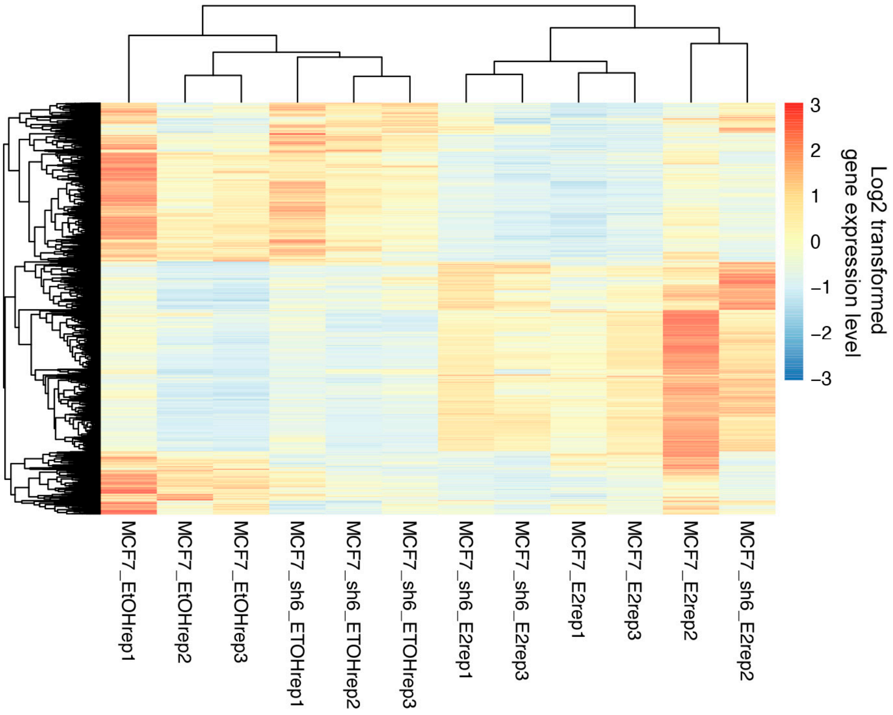

Figure S4. Transcriptomic Changes induced by TRIM33 Knockdown. MCF-7/shCtrl (**MCF7**) and MCF-7/shTRIM33#6 (**MCF7_sh6**) cells were treated in triplicate with 0.1% ethanol (EtOH) or 1 nM E2 for 24 h. Extracted, ribo-depleted RNA was used for sequencing. The top 10% of genes that were significantly differentially expressed (q≥0.05, abs(log2FC)≥0.7) between samples are shown as normalized expression counts.

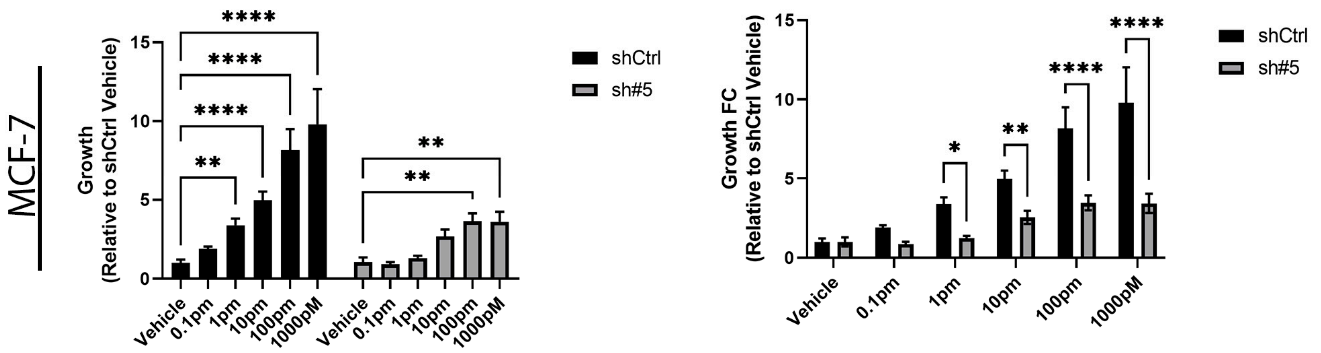

Figure S5. TRIM33 Knockdown with Separate Construct (#5) Suppresses E2-Stimulated Growth. MCF-7/shCtrl and MCF-7/shTRIM33#5 cells were hormone-deprived for 4 d prior to treatment with 0-1 nM E2. Cells were analyzed 7 d later by SRB assay. Data are shown as mean of triplicates + SD. *$p \leq 0.05$, **$p \leq 0.01$, ***$p \leq 0.001$, ****$p \leq 0.0001$ by Bonferroni multiple comparison-adjusted post-hoc test. Left panel depicts growth relative to vehicle-treated shCtrl (A) or Luc (B) cells. Right panel depicts growth relative to each vehicle-treated control.

Table S1. Primers for RT-qPCR

Gene	Sequence
AREG FWD	5′tga tcc tca cag ctg ttg ct 3′
AREG REV	5′ tcc att ctc ttg tcg aag ttt ct 3′
PDZK FWD	5′ gtccgggtggttgagaagt 3′
PDZK1 REV	5′ ttgatcctaagaactctgtctcca 3′
PR FWD	5′gat tca gaa gcc agc cag ag 3′
PR REV	5′ tgc ctc tcg cct agt tga tt 3′
IRS1 FWD	5′gca acc aga gtg cca aag tga 3′
IRS1 REV	5′ gga gaa agt ctc gga gct atg 3′
β-actin FWD	5′ tga cag gat gca gaa gga gat 3′
β-actin REV	5′ gcg ctc agg agg agc aat 3′

Table S2. Primers for Generation of Flag-Turbo-ESR1 and TRIM33/OVEXP Plasmid Constructs

Primer	Sequence
(Flag-Turbo-Control) Lenti-Turbo Fwd	5′ agggggatccaccggttcgatggactacaaagaccatga 3′
(Flag-Turbo-Control) Turbo-Lenti Rev	5′ tgcggtctgccgaaaagtagacccagctttcttgtacaaa 3′
(Flag-Turbo-ER) Turbo-ESR1 FWD	5′ gtctgcggtctgccgaaaagggtggaggcggttctaccat 3′
(Flag-Turbo-ER) ESR1-Lenti Rev	5′ gtttccctgccacagtctagacccagctttcttgtacaaa 3′
(Flag-Turbo-ER) Turbo-ESR1 Rev	5′ gtctgcggtctgccgaaaagggtggaggcggttctaccat 3′
(Flag-Turbo-ER) Turbo-ESR1 FWD	5′ gtctgcggtctgccgaaaagGgtggaggcggttctaccat 3′
(TRIM33 OVEXP) TRIM33 OVEXP FWD	5′ gatccaccggttcgGCCACCatggcggaaaacaaaggcgg 3′
(TRIM33 OVEXP) TRIM33 OVEXP REV	5′ gaccagtacatataaagtaaacccagctttcttgtacaaa 3′
