# Supplementary figures and images for: TRIM33 Is a Co-Regulator of Estrogen Receptor Alpha
Source: Cancers (Basel). 2024 Feb 20;16(5):845. doi: 10.3390/cancers16050845 (PMC10930732; doi:10.3390/cancers16050845)

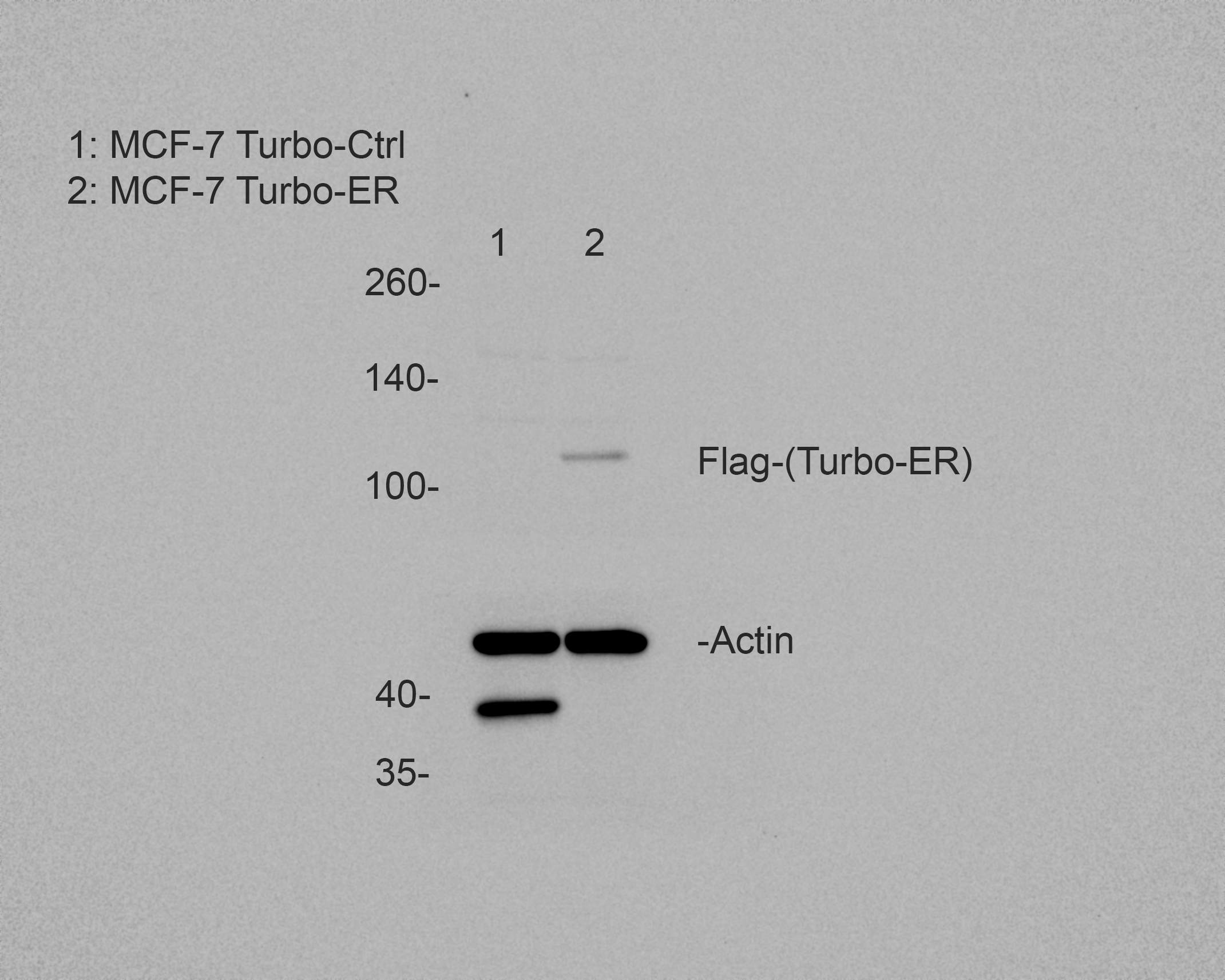

Supplement: Supplementary file 1 [file cancers-16-00845-s001.zip › cancers-2855756-File S1/Figure 1A (left) estrogen-induced proximity labeling of ER interactors (longer exposure).png]

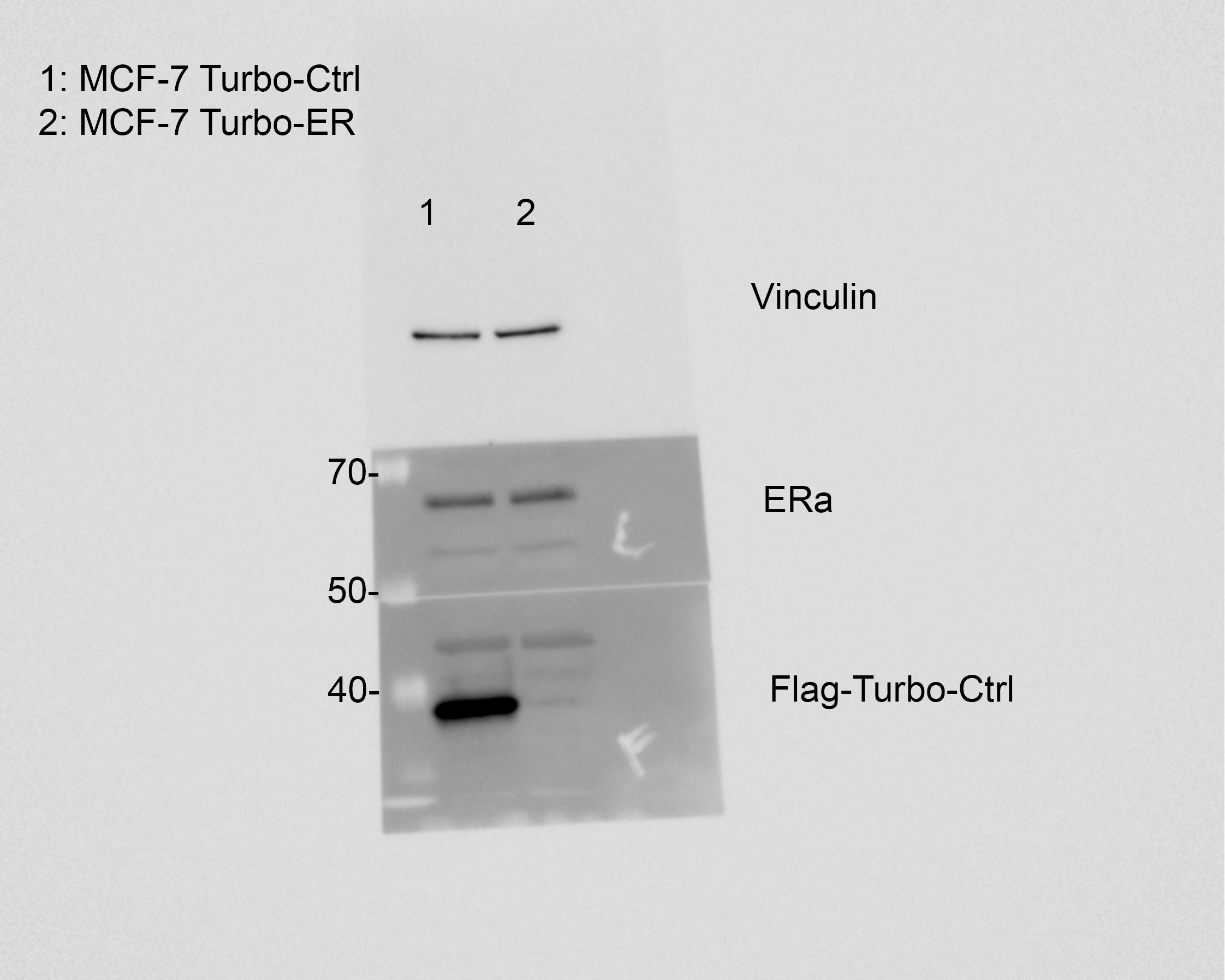

Supplement: Supplementary file 1 [file cancers-16-00845-s001.zip › cancers-2855756-File S1/Figure 1A (left) estrogen-induced proximity labeling of ER interactors (shorter exposure).png]

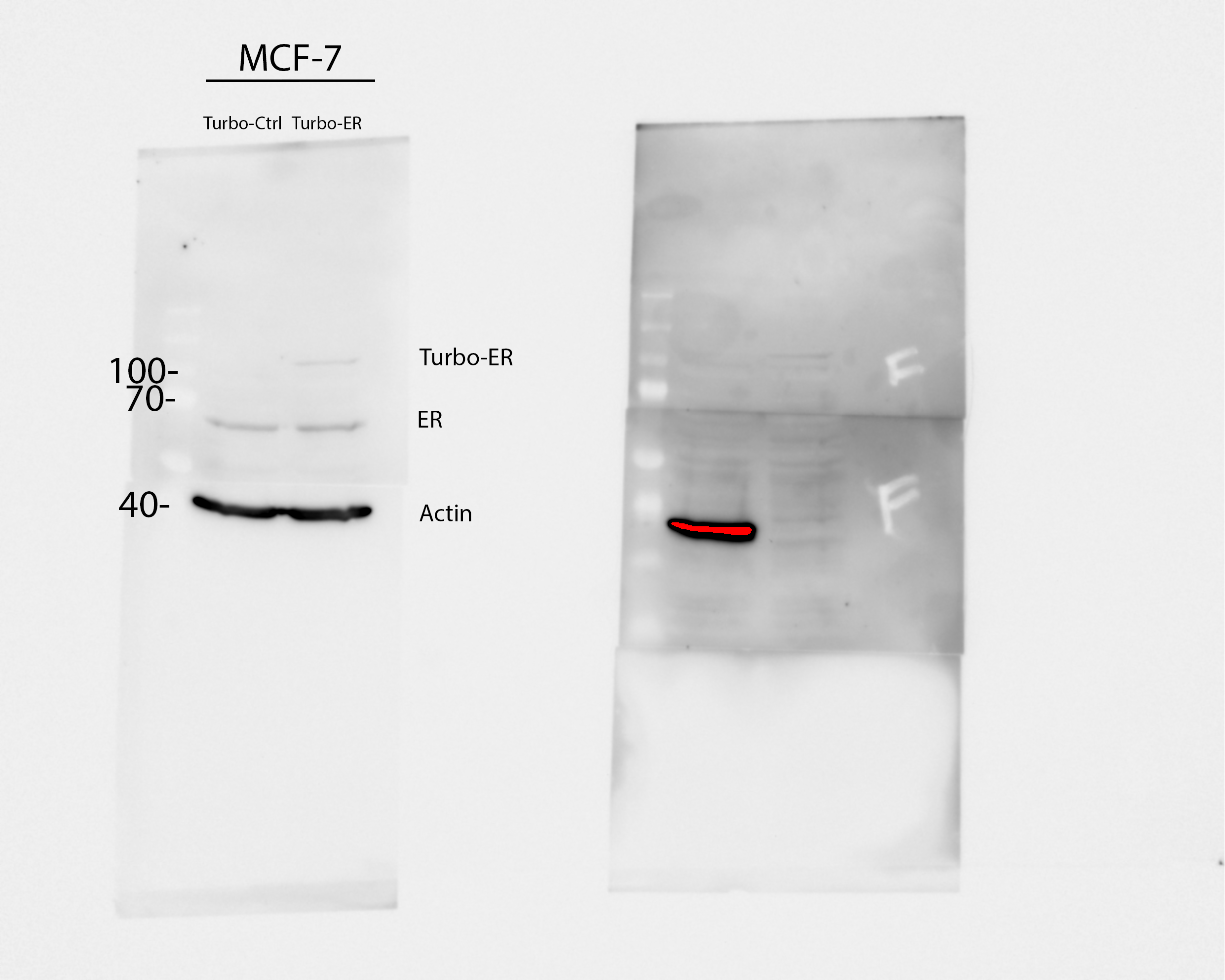

Supplement: Supplementary file 1 [file cancers-16-00845-s001.zip › cancers-2855756-File S1/Figure 1A (right) Estrogen induced proximity labeling of ER Interactors.png]

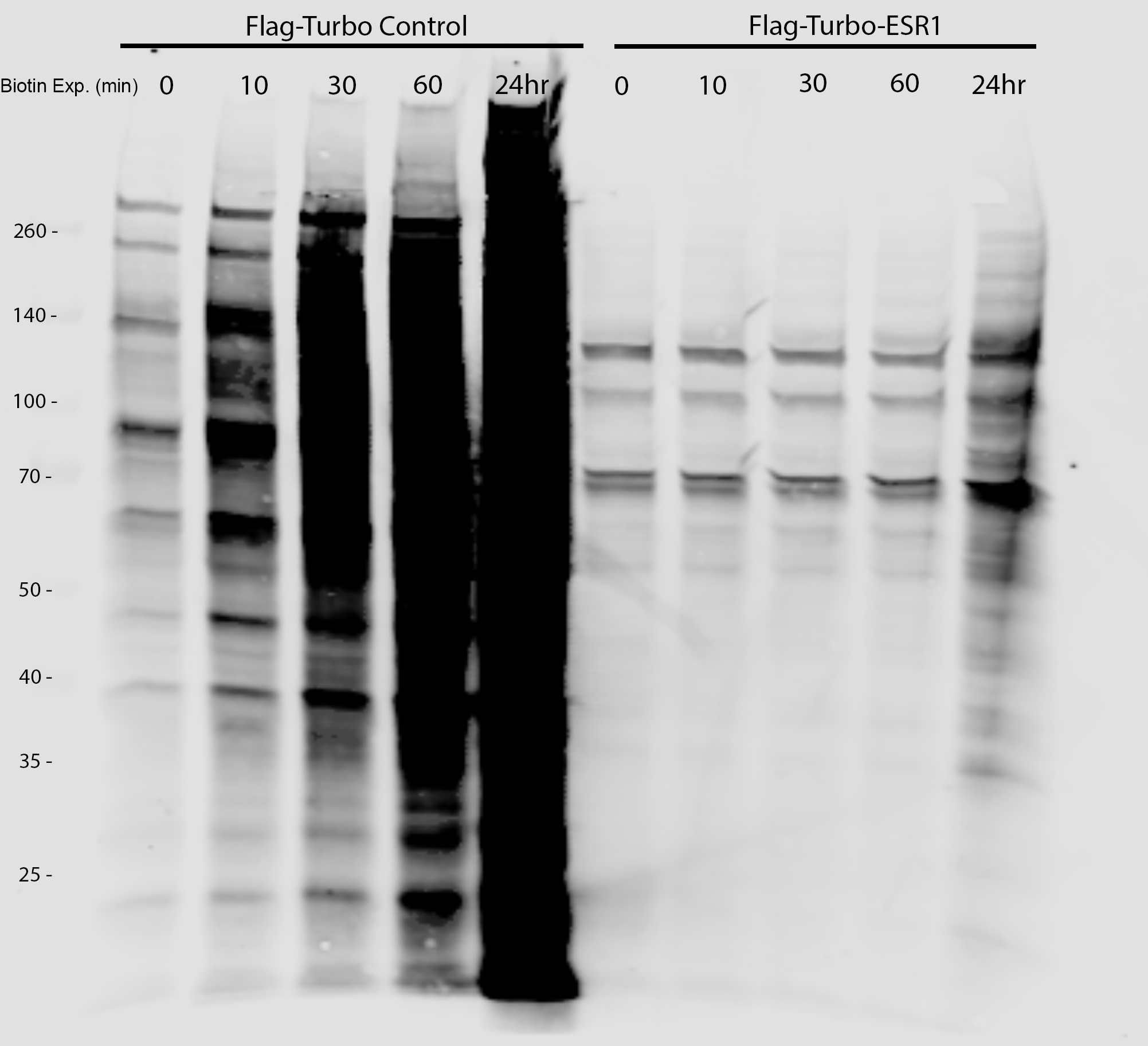

Supplement: Supplementary file 1 [file cancers-16-00845-s001.zip › cancers-2855756-File S1/Figure 1B- Estrogen induced Proximity labeling of the ER interactors.png]

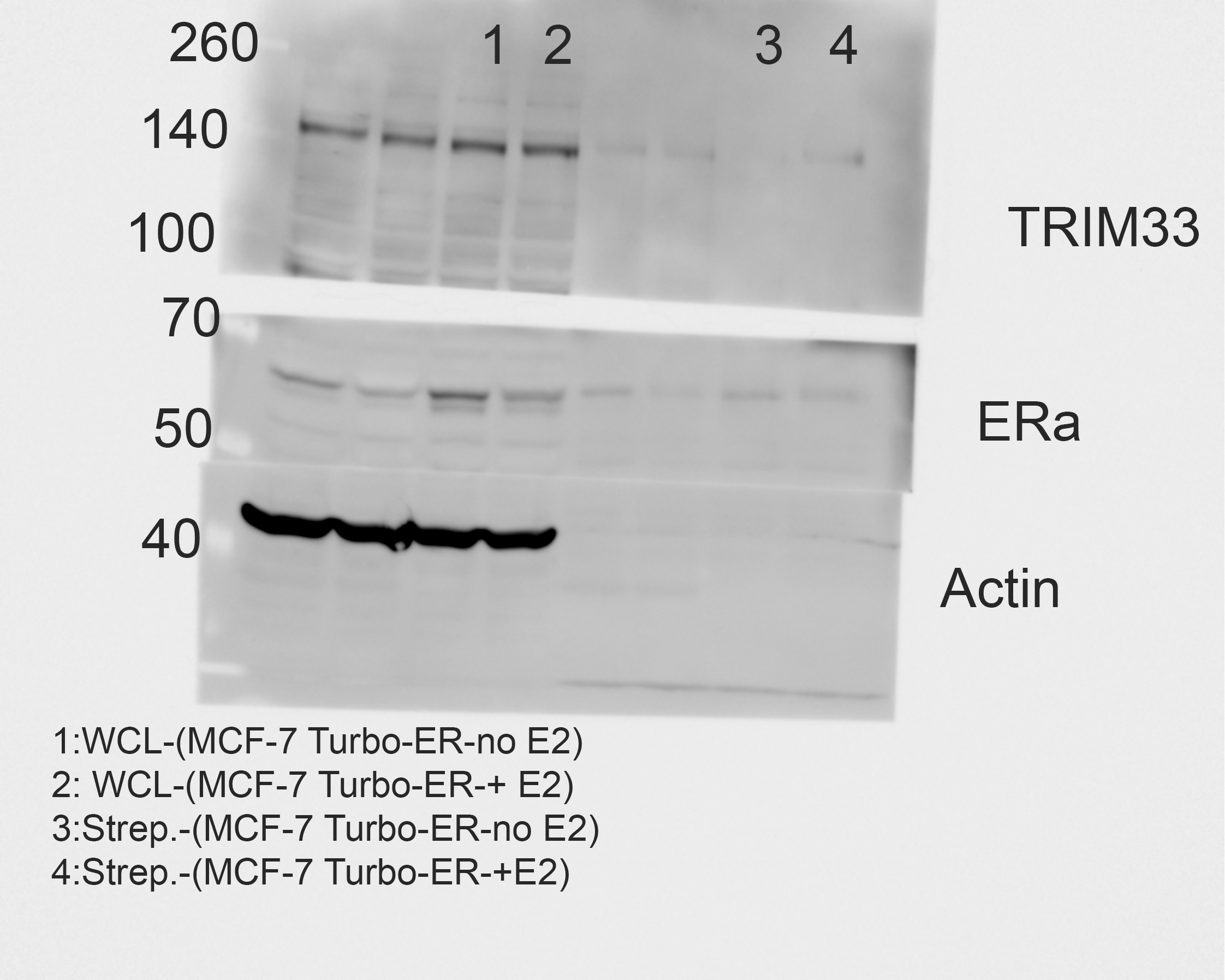

Supplement: Supplementary file 1 [file cancers-16-00845-s001.zip › cancers-2855756-File S1/Figure 1E (left)- Estrogen Induced Proximity labeling of ER interactors (TRIM,ER, long exposure).png]

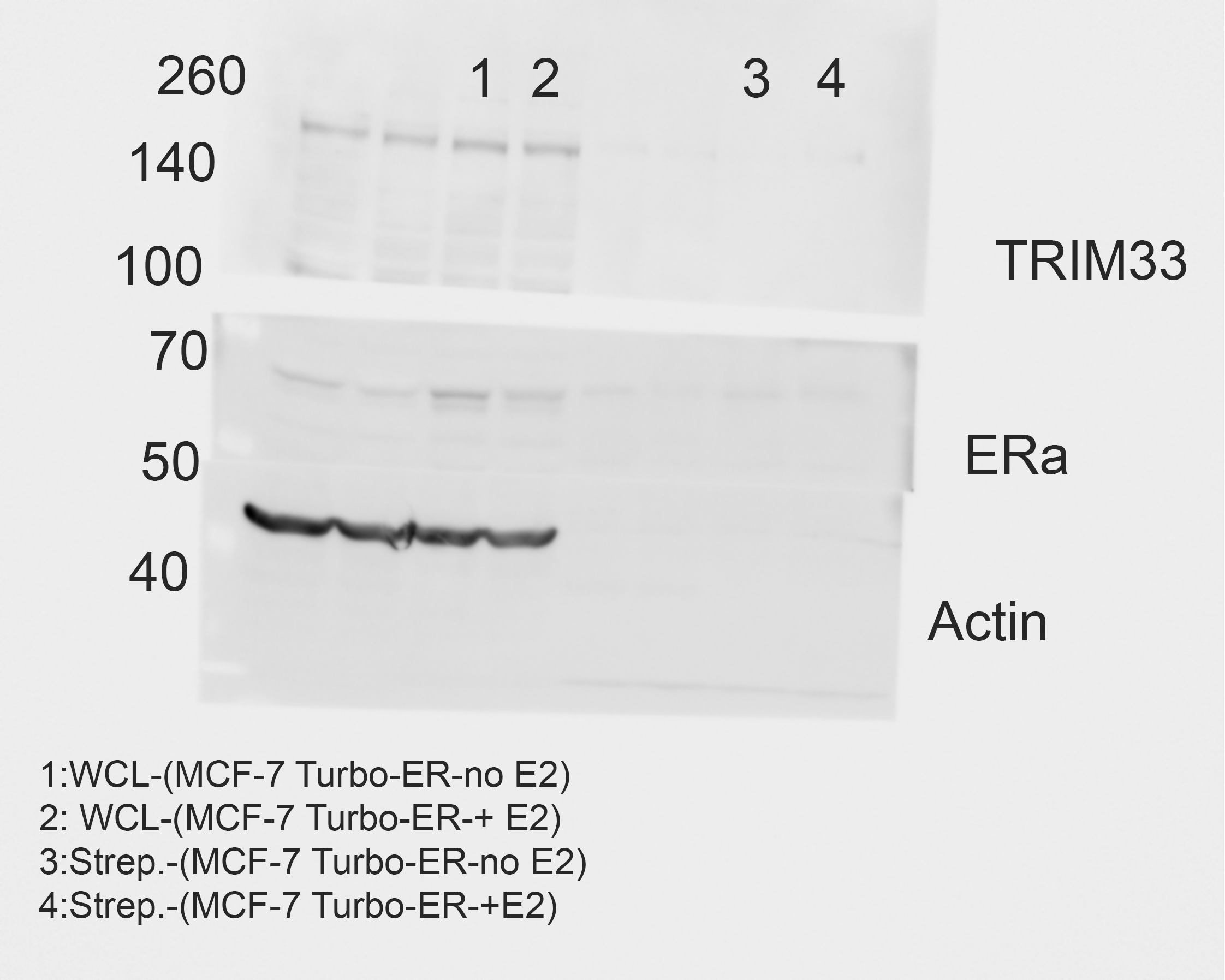

Supplement: Supplementary file 1 [file cancers-16-00845-s001.zip › cancers-2855756-File S1/Figure 1E (left)- Estrogen Induced Proximity labeling of ER interactors (TRIM,ER, short exposure).png]

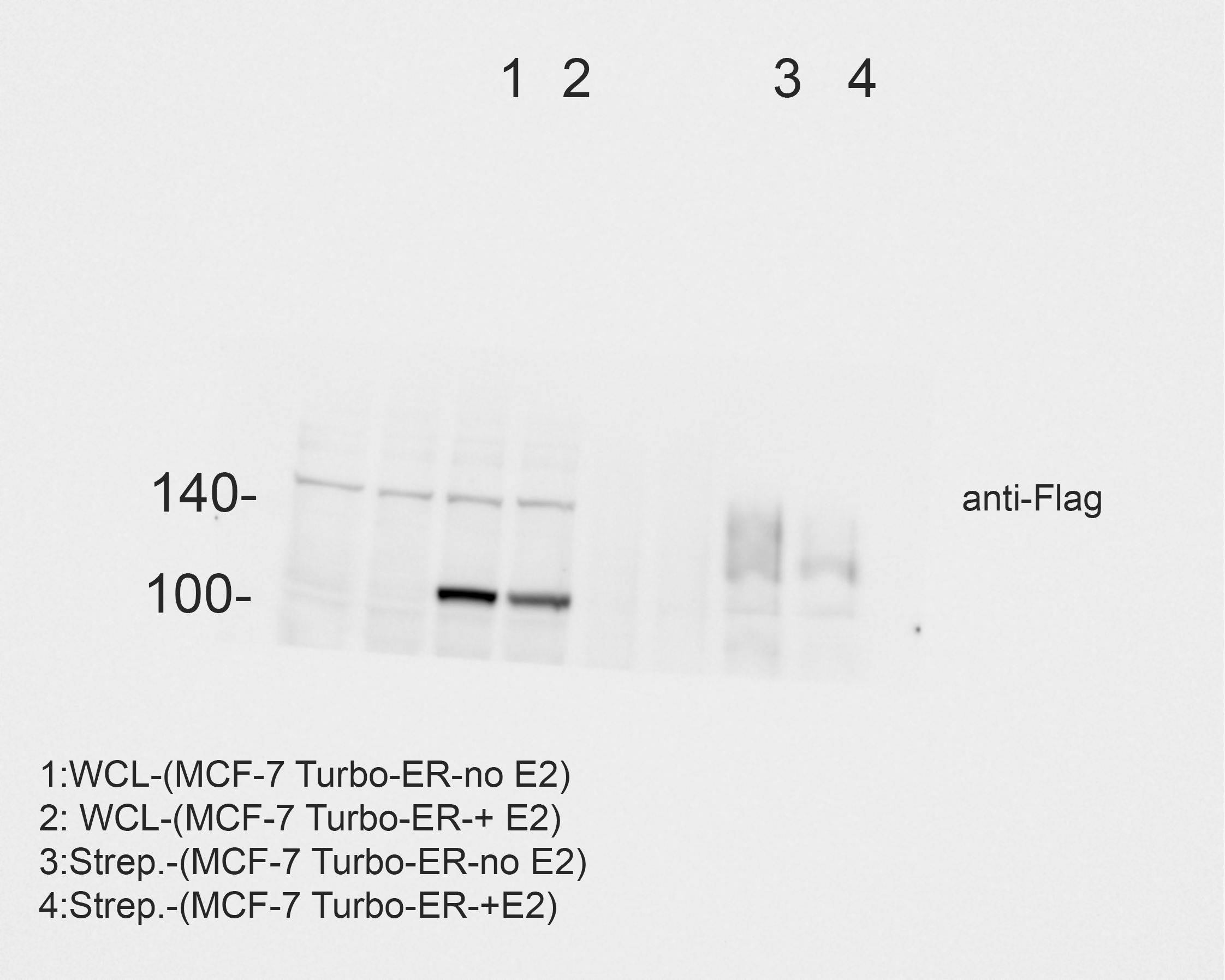

Supplement: Supplementary file 1 [file cancers-16-00845-s001.zip › cancers-2855756-File S1/Figure 1E (right)- Estrogen Induced Proximity labeling of ER interactors (FLAG).png]

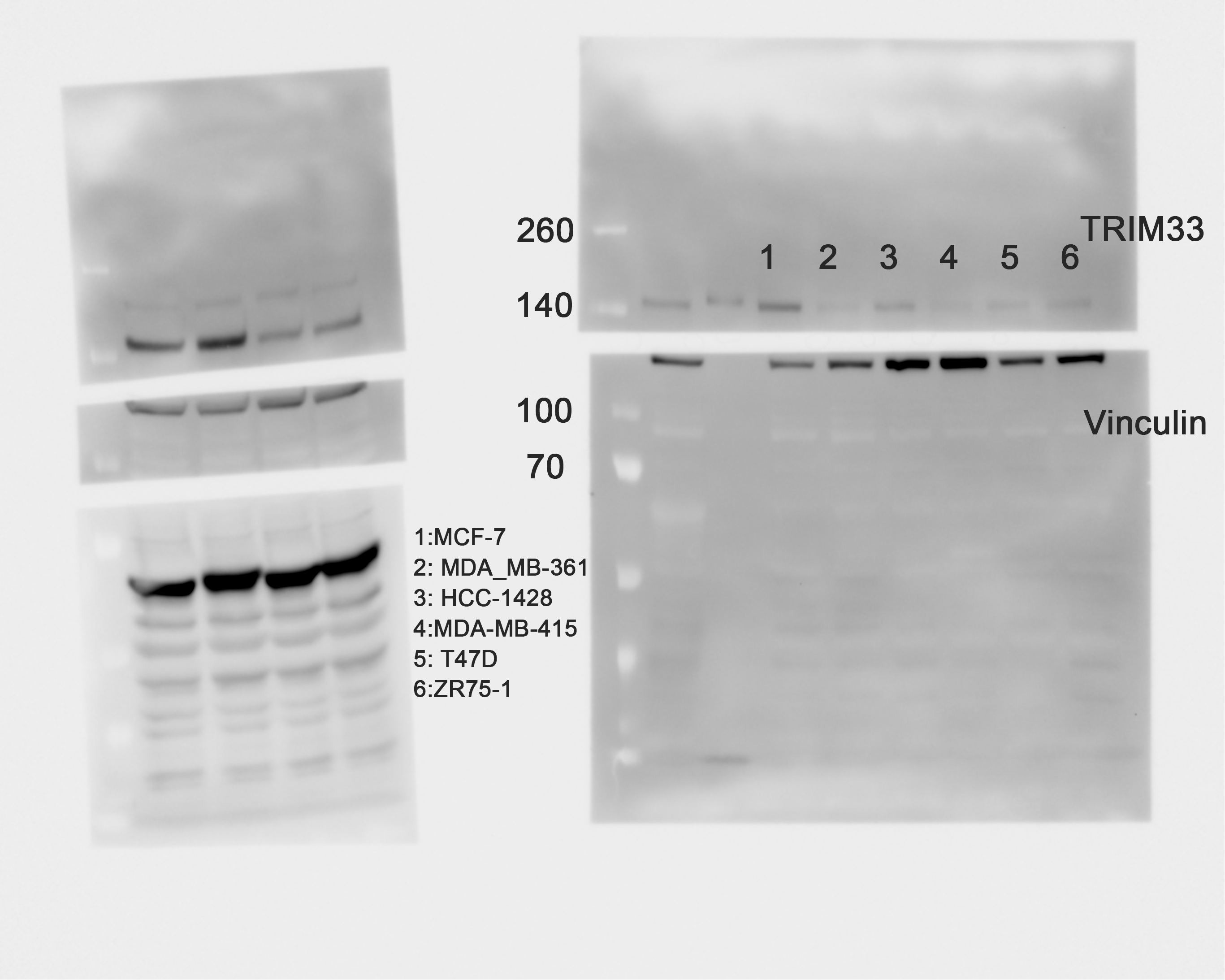

Supplement: Supplementary file 1 [file cancers-16-00845-s001.zip › cancers-2855756-File S1/Figure 2A TRIM33 regulates ER signaling.png]

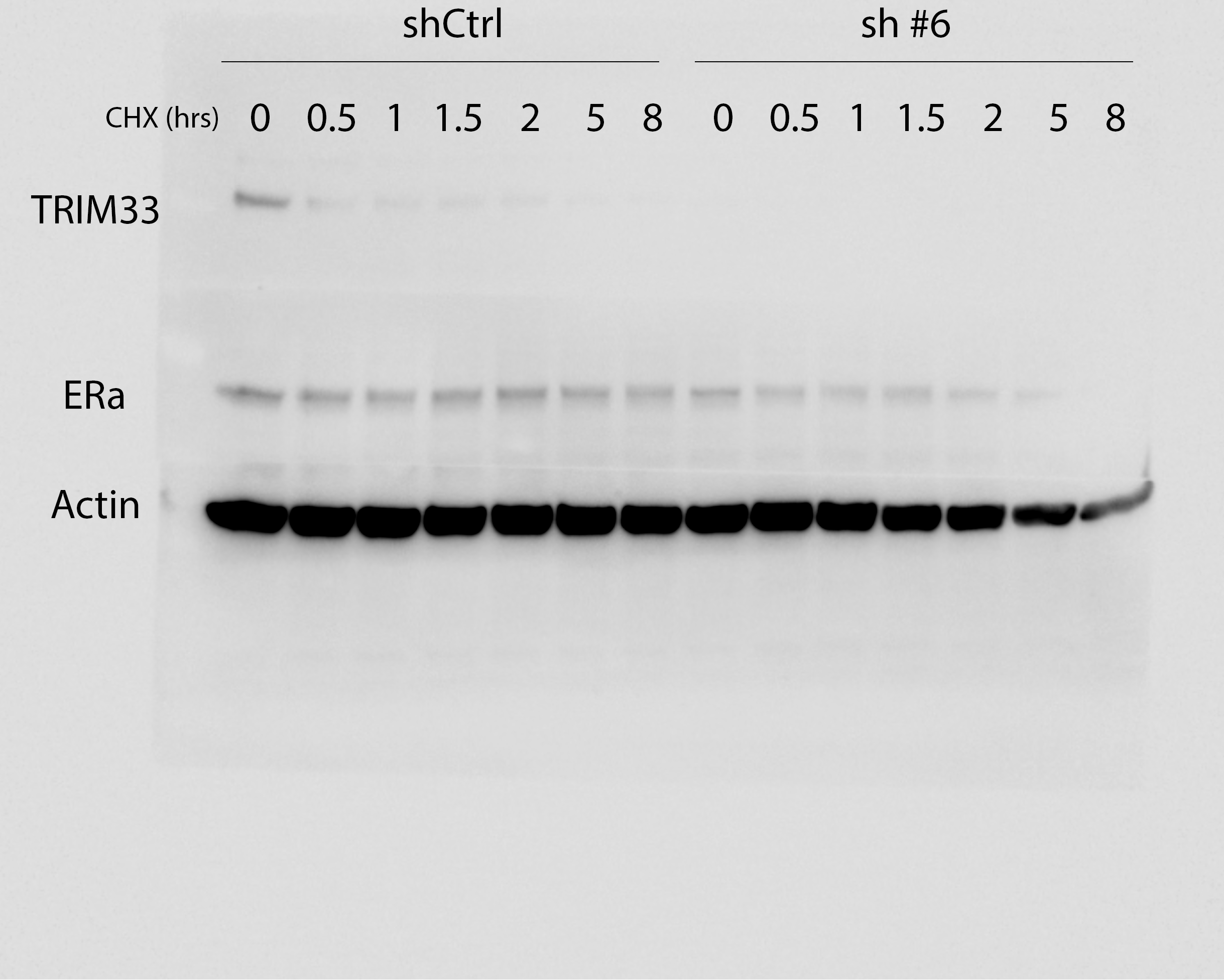

Supplement: Supplementary file 1 [file cancers-16-00845-s001.zip › cancers-2855756-File S1/Figure 4A- TRIM33 Stabilizes ER levesl MCF-7.png]

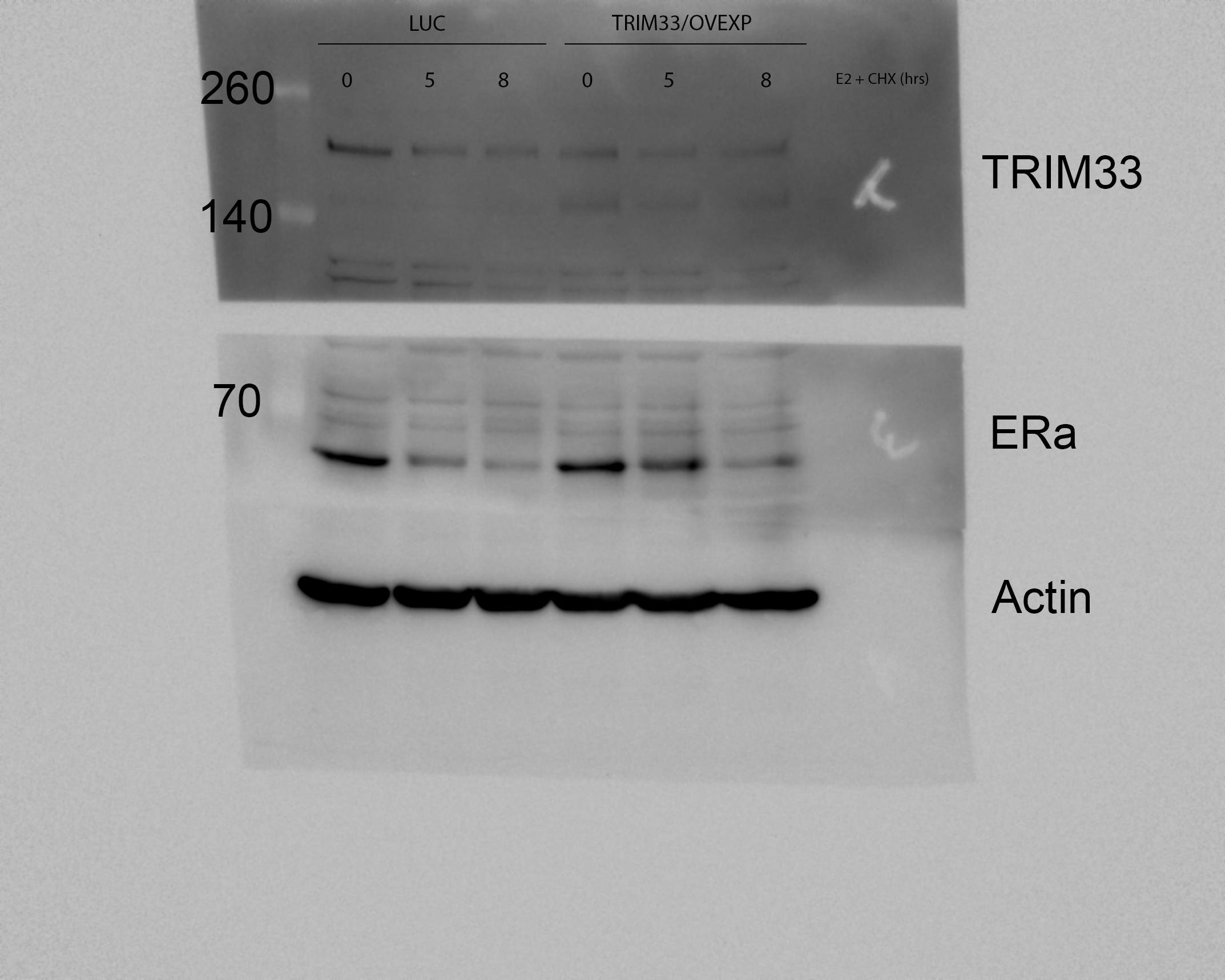

Supplement: Supplementary file 1 [file cancers-16-00845-s001.zip › cancers-2855756-File S1/Figure 4B- TRIM33 Stabilizes ER Levels T47D.png]

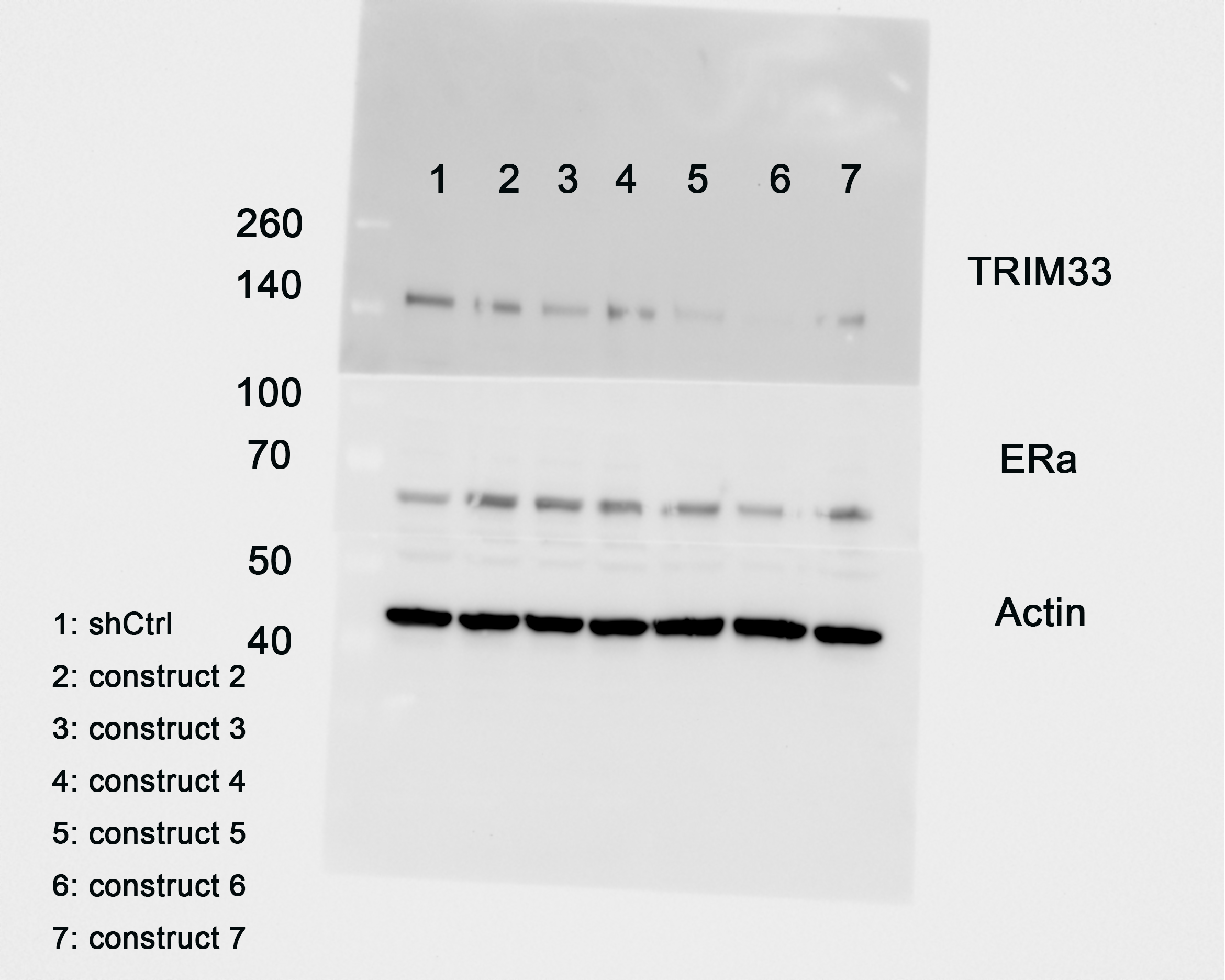

Supplement: Supplementary file 1 [file cancers-16-00845-s001.zip › cancers-2855756-File S1/Supplemental figure S2 Generation of TRIM33 shRNA Knockdown cell lines.png]

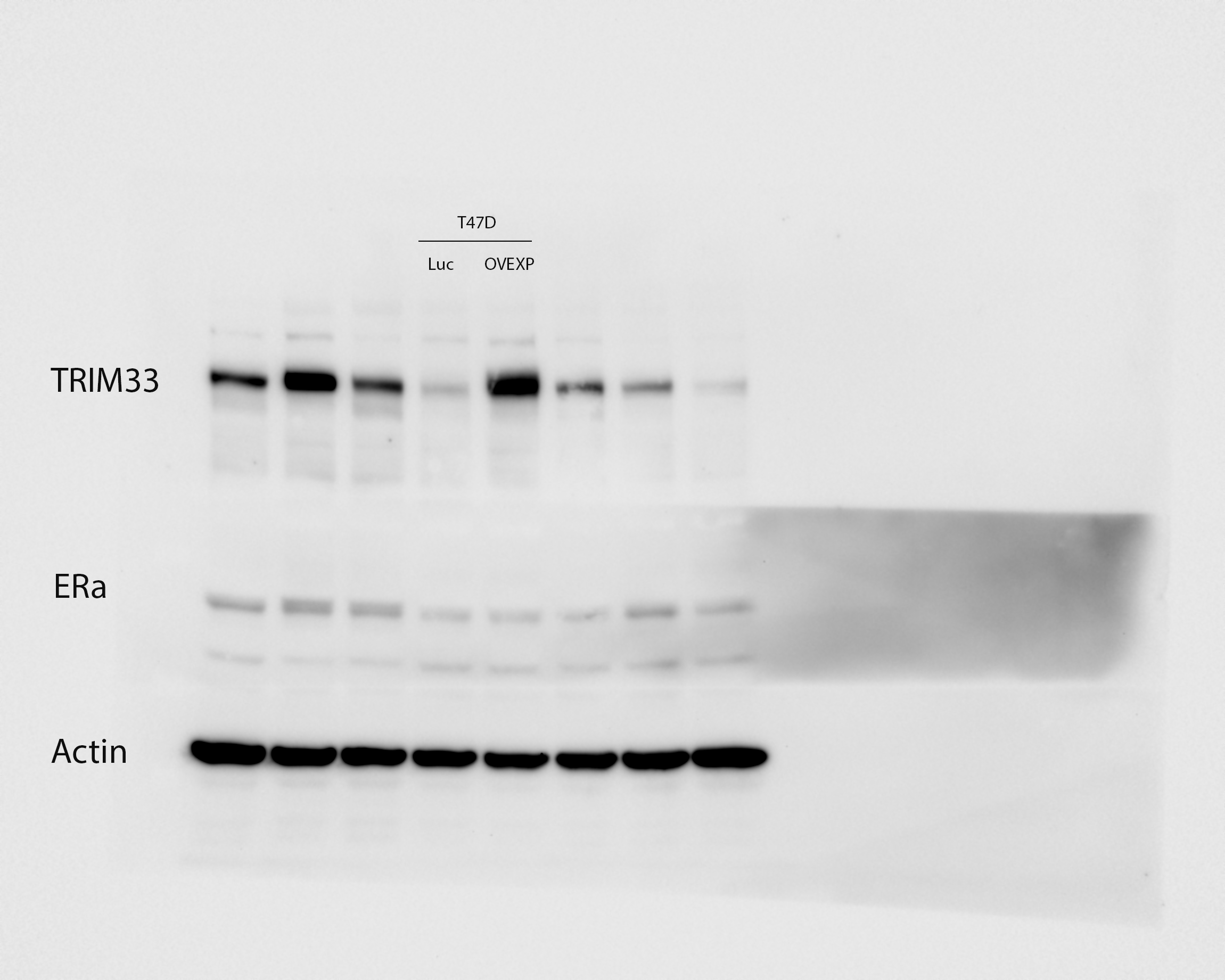

Supplement: Supplementary file 1 [file cancers-16-00845-s001.zip › cancers-2855756-File S1/Supplemental figure S3 (left)- Generation of TRIM33 -overexpressing cell lines T47D.png]

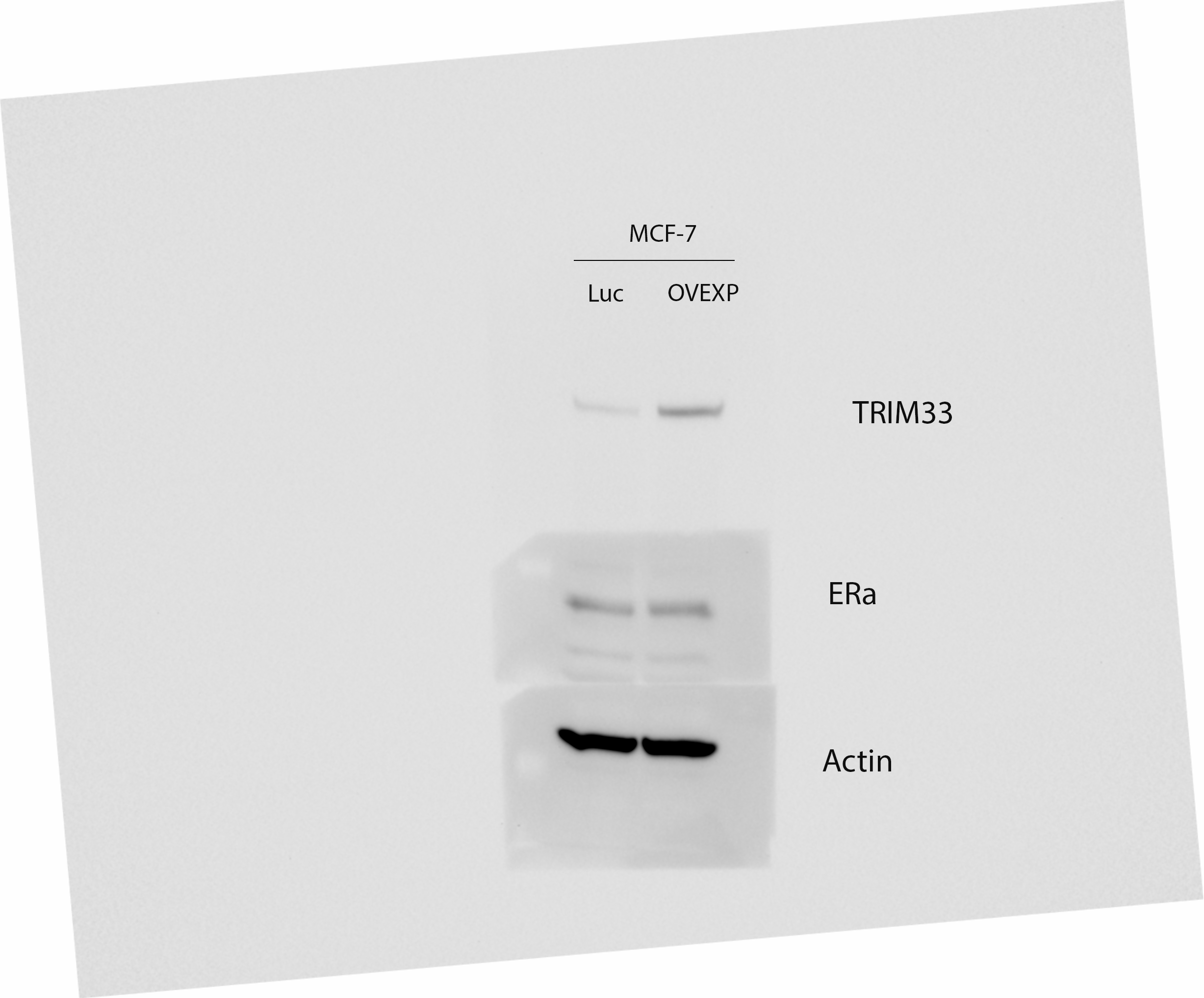

Supplement: Supplementary file 1 [file cancers-16-00845-s001.zip › cancers-2855756-File S1/Supplemental figure S3 (right)- Generation of TRIM33 overexpressing cell lines MCF-7.png]
